# The influence of particle activated carbon on heavy metal passivation and antibiotic degradation in various organic fertilizers

**DOI:** 10.3389/fmicb.2025.1686052

**Published:** 2025-09-19

**Authors:** Zifang Chi, Yuru Li, Huai Li

**Affiliations:** ^1^Key Lab of Groundwater Resources and Environment, Ministry of Education, Jilin University, Changchun, China; ^2^Northeast Institute of Geography and Agroecology, Chinese Academy of Sciences, Changchun, China

**Keywords:** particle activated carbon, heavy metal passivation, antibiotic degradation, organic fertilizer, influence

## Abstract

This study investigated the effects of different particle sizes of activated carbon [small particle activated carbon (S-AC, 2–4 mm), medium particle activated carbon (M-AC, 3–6 mm), and large particle activated carbon (B-AC, 5–8 mm)] on the physicochemical properties of organic fertilizers (chicken manure, sheep manure, cow manure and pig manure), the passivation of heavy metals, and the degradation of antibiotics. The results showed that the addition of particle activated carbon could increase the pH value of organic fertilizers, reduce the cation exchange capacity (CEC) value, and its fragmentation led to an increase in organic matter in organic fertilizers, but had no significant effect on the electrical conductivity (EC) value. The small particle activated carbon (S-AC) had high mechanical strength (compressive strength 4.014 MPa), low loss rate, and high recovery rate, showing the best remediation performance. After adding S-AC, the removal rates of total copper (Cu) and zinc (Zn) in organic fertilizers reached 23.71–28.57% and 15.41–17.81% respectively, and the maximum passivation rates of exchangeable fraction Cu and Zn were 61.31 and 29.10%. At the same time, S-AC significantly promoted the degradation of antibiotics, with the degradation rates of tetracycline (TC) and ciprofloxacin (CIP) reaching 81.38–85.81% and 76.53–80.59% within 30 days.

## 1 Introduction

Livestock and poultry farming is an important economic pillar industry in Chinese agriculture. With the rapid growth of meat product supply, Chinese livestock and poultry farming industry has developed rapidly. Traditional farming methods have been gradually reduced, and farming methods have been gradually transformed toward large-scale and intensive. This has led to a sharp increase in the quantity and output of livestock and poultry farming, as well as an increase in the amount of livestock and poultry manure emissions (Zilio et al., 2020; Liu et al., 2022). To accelerate the growth of livestock and poultry and enhance their immunity, livestock feed often contains heavy metals and antibiotic drugs (Zhou et al., 2016; Hashemi, 2018). Livestock and poultry have extremely low utilization rates of heavy metals and antibiotics, and heavy metals and antibiotics residues still exist in livestock and poultry manure (Cha and Carlson, 2019; Zhang et al., 2021; Gaballah et al., 2021). Antibiotics and heavy metals enter the environment through unhygienic treatment of livestock and poultry manure for agricultural use, causing widespread drug resistance in environmental microorganisms. This not only harms the health functions of farmland soil, but also poses great risks to the ecological environment and human health (Kuppusamy et al., 2018; Zhen et al., 2020; Bai et al., 2019).

The main method for handling agricultural waste such as livestock manure in our country is to carry out composting treatment (Kim et al., 2018). After composting fermentation, the proportion of heavy metals with strong mobility and toxicity will be significantly reduced, and they will transform into more stable forms such as oxidized state and residue state, thereby causing the passivation of heavy metals and the reduction of toxicity. At the same time, antibiotics are degraded under the action of microorganisms, and the residual amount of antibiotics in the feces is significantly reduced (Awasthi et al., 2020; Zheng et al., 2022; Ezzariai et al., 2018). Although the composting method can reduce the concentration of antibiotics and the activity of heavy metals to a certain extent, the effect of composting treatment is limited. Especially during the composting process, heavy metals do not undergo transfer and are accompanied by decomposition of gasses, water, and organic substances. The volume and weight of the compost decrease, and the material concentration shows a “relative concentration effect of heavy metals” (Chen et al., 2017). Therefore, it is not sufficient to solely rely on composting to reduce antibiotics and to neutralize heavy metals.

Studies have shown that the application of passivators can effectively promote the metal passivation and antibiotic reduction in composting (Chen et al., 2025; Abdellah et al., 2022). Among them, biochar, which has the characteristics of stability, numerous micropores, and strong adsorption capacity, is widely used. Currently, both domestically and internationally, there are more and more studies on using biochar as a passivator to reduce secondary pollution and accelerate composting decomposition in composting (Chen et al., 2022; Wang et al., 2023; Awasthi et al., 2021). Chen et al. (2010) added bamboo charcoal to fecal compost and achieved effective immobilization of copper and zinc. The contents of available copper (Cu) and zinc (Zn) in the compost products were significantly reduced, with immobilization rates reaching 65 and 35%, respectively. Awasthi et al. (2020) utilized biochar to enhance pig manure composting, effectively immobilizing heavy metals (Cu and Zn). They found that the content of heavy metals was significantly correlated with microbial abundance, with actinomycetes (56.22%) and proteobacteria (35.40%) being the dominant groups. Zhou et al. (2023) investigated the immobilization effect of Cu and Zn in biochar-enhanced livestock and poultry manure composting. The results indicated that 5 and 10% BC were beneficial for the immobilization of Cu and Zn, respectively. BC promoted the immobilization of Cu and Zn by accelerating the production of HA, optimizing the abundance of Firmicutes, and increasing the pH of the compost. Lin et al. (2021) used a composite material of zeolite and activated carbon as an additive for pig manure composting. The results showed that the composite additive could accelerate the immobilization of heavy metals through surface adsorption and precipitation, and also accelerate the attenuation of antibiotics in the short term. Yang et al. (2022) utilized biochar to promote the immobilization of heavy metals (Zn, Cu) and the reduction of antibiotics (enrofloxacin, sulfamethoxazole) in livestock and poultry manure. The results indicated that biochar facilitated electron transfer among microorganisms and significantly reduced the environmental risk of co-contamination by heavy metals and antibiotics.

In the existing studies, the biochar is mostly in the form of small particle powders, with low mechanical strength and prone to fragmentation. After composting, it is difficult to separate and recycle the additives. Although the migration and transformation of heavy metals and the reduction of biological effectiveness occur in the composting materials, heavy metals and some antibiotics still remain in the composting materials. Based on this, this study innovatively uses particle activated carbon as an additive and systematically examines the effects of different particle sizes of activated carbon on the metal passivation and antibiotic degradation of various organic fertilizers. Through the organic fertilizer remediation experiments, the dynamic changes in the physicochemical properties, metal forms, and antibiotic contents of the organic fertilizers during the particle activated carbon remediation process are revealed, and the optimal particle size of the activated carbon is determined, aiming to provide new technical solutions and theoretical support for the control of heavy metal and antibiotic pollution in organic fertilizers.

## 2 Materials and methods

### 2.1 Test materials

**Figure 1:**
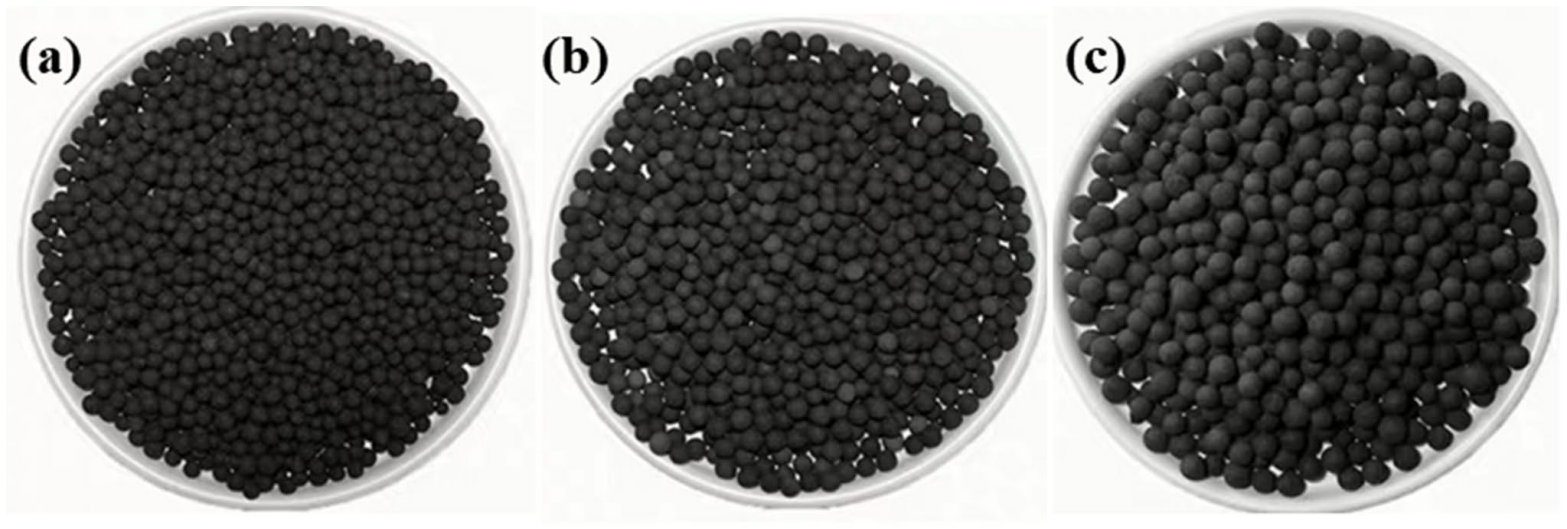
Activated carbon: **(a)** S-AC; **(b)** M-AC; **(c)** B-AC.

Chicken manure fertilizer (J), pig manure fertilizer (Z), sheep manure fertilizer (Y), and cow manure fertilizer (N) are all commercial products. They are naturally air-dried, then ground by a grinder and sieved for use. The particle activated carbon (AC) (Figure 1) used is purchased from Henan Lvyuan Activated Carbon Co., Ltd. The small particle activated carbon (2–4 mm), medium particle activated carbon (3–6 mm), and large particle activated carbon (5–8 mm) are, respectively, labeled as S-AC, M-AC, and B-AC. The physical and chemical properties of the relevant materials are shown in Table 1.

**Table 1:** Main physical and chemical properties of experimental materials.

Group	pH	EC (mS/cm)	Moisture content (%)	TOC (%)	Total_Zn_ (mg/kg)	Total_Cu_ (mg/kg)
J	7.33	4.17	17.25	22.74	706.75	253.54
Z	7.97	2.30	25.75	22.56	164.24	54.88
Y	7.21	3.81	18.13	22.43	698.08	242.58
N	7.36	4.21	17.58	17.52	734.53	248.80
S-AC	8.53	1.71	3.67	51.95	39.03	-
M-AC	8.63	1.20	3.13	52.80	29.53	-
B-AC	10.13	3.86	5.21	53.34	45.00	27.95

- indicates no detection; The initial organic fertilizer did not test positive for TC and CIP.

### 2.2 Experiment reagents

Zinc sulfate heptahydrate (ZnSO_4_·7H_2_O), concentrated sulfuric acid (H_2_SO_4_), concentrated hydrochloric acid (HCl), concentrated nitric acid (HNO_3_), perchloric acid (HClO_4_) were purchased from Beijing Chemical Factory; hydrofluoric acid (HF), acetic acid (HAc), hydroxylamine hydrochloride (NH_2_OḤHCl), ammonium acetate (NH_4_OAc), citric acid (C_6_H_8_O_7_·H_2_O), ethylenediaminetetraacetic acid disodium salt (Na_2_EDTA·2H_2_O) were purchased from Tianjin Huihang Chemical Technology Co., Ltd.; trichloro hexammine cobalt (Co(NH_3_)_6_Cl_3_), tetracycline, ciprofloxacin were purchased from Shanghai Aladdin Biochemical Technology Co., Ltd.; triethanolamine (C_6_H_15_NO_3_), disodium hydrogen phosphate (Na_2_HPO_4_·12H_2_O) were purchased from Tianjin Yongda Chemical Reagent Co., Ltd.; methanol, acetonitrile were purchased from Shandong Yuwang Hetai New Materials Co., Ltd.; cupric chloride dihydrate (CuCl_2_·2H_2_O) was purchased from Tianjin Yongsheng Fine Chemical Co., Ltd.; potassium dichromate (K_2_Cr_2_O_7_) was purchased from Tianjin Xintong Fine Chemical Co., Ltd.; mercuric sulfate was purchased from Liaoning Quanrui Reagent Co., Ltd.; glucose (C_6_H_12_O_6_) was purchased from Tianjin Fucheng Chemical Reagent Co., Ltd.; hydrogen peroxide solution (H_2_O_2_) was purchased from Tianjin Xinpeng Chemical Co., Ltd.; ethylenediaminetetraacetic acid disodium salt (C_14_H_23_N_3_O_10_) was purchased from Tianjin Kemiou Chemical Reagent Co., Ltd.; anhydrous calcium chloride (CaCl_2_) was purchased from Tianjin Kaitong Chemical Reagent Co., Ltd.; hydrated oxalic acid (C_2_H_2_O_4_·2H_2_O) was purchased from Tianjin Guangfu Technology Development Co., Ltd. Methanol and acetonitrile are chromatographic grade, and the remaining reagents are analytical grade.

### 2.3 Configuration of polluted fertilizers

Add 200 g of sieved reserve organic fertilizer to a 500 mL polyethylene bottle. Measure and prepare high-concentration copper and zinc contaminated solutions (10 g/L) using CuCl_2_·2H_2_O and ZnSO_4_·7H_2_O, respectively. Set the Cu contamination concentration of the organic fertilizer at 800 mg/kg; the Zn contamination concentration at 1500 mg/kg. Add the Cu and Zn contaminated solutions to the organic fertilizer in accordance with the initial Cu and Zn concentrations of each type of organic fertilizer, and add pure water to ensure that each group of organic fertilizer has a moisture content of 60%. Mix well and stabilize for 1 month. Spray high-concentration tetracycline and ciprofloxacin mother solutions onto the organic fertilizer. Set the tetracycline TC contamination concentration of the organic fertilizer at 100 mg/kg; the ciprofloxacin CIP contamination concentration at 100 mg/kg. Let it stabilize for 24 h.

### 2.4 Experimental design

#### 2.4.1 Remediation experiment design

Different particle sizes of activated carbon were added to different types of organic fertilizers (with the carbon addition amount accounting for 10% of the dry weight of the organic fertilizer), and the mixture was placed on a shaking oscillator for a 30-day remediation experiment. Samples were taken at 0 d, 2 d, 6 d, 12 d, 20 d, and 30 d, and the particle activated carbon was separated from the organic fertilizer. The changes in the physical and chemical properties of the organic fertilizer, as well as the levels of heavy metals and antibiotics, were recorded. A total of four experimental groups were designed in the study. All the experiments are in triplicate. The parameters of each group are detailed in Table 2.

**Table 2:** Parameter settings for the experimental group.

Group	Configuration	Notes
J	No AC added	J-CK
	Add 10% S-AC	J-S
	Add 10% M-AC	J-M
	Add 10% B-AC	J-B
Y	No AC added	Y-CK
	Add 10% S-AC	Y-S
	Add 10% M-AC	Y-M
	Add 10% B-AC	Y-B
N	No AC added	N-CK
	Add 10% S-AC	N-S
	Add 10% M-AC	N-M
	Add 10% B-AC	N-B
Z	No AC added	Z-CK
	Add 10% S-AC	Z-S
	Add 10% M-AC	Z-M
	Add 10% B-AC	Z-B

In this study, the exchangeable fraction was used as the measurement indicator for the metal passivation effect. The formula for calculating the passivation rate is as follows:


$$Q=\frac{X_{0}-X_{1}}{X_{0}}*100\%$$

In the formula, *Q* represents the passivation rate (%). X_0_ is the initial content of the exchangeable fraction of heavy metals (mg/kg). X_1_ is the content of the exchangeable fraction of heavy metals at the end of the process (mg/kg).

The antibiotic content of samples from different repair periods was fitted using the first-order degradation kinetic equation, and the formula is as follows:


$$C_{t}=C_{0}(e^{-\mathrm{kt}})$$

In the formula, K represents the degradation rate constant (d^−1^). The half-life of the antibiotic can be calculated using the formula T_1/2_ = ln2/K, where T_1/2_ is the half-life (d).

#### 2.4.2 Determination of AC loss rate

The loss rate is an indicator for measuring the mechanical strength of the material. Generally, the lower the loss rate, the higher the mechanical strength. Take 3 g of particle activated carbon and place it in a 50 mL centrifuge tube. Add 30 mL of ultrapure water. Shake at room temperature for 0.5, 1, 2, 4, 6, 12, 24, 48, and 72 h, respectively. Filter the activated carbon and dry it at 70 °C until it reaches a constant weight. All the experiments are in triplicate.

The calculation of the loss rate is as follows:


$$P=\frac{M_{0}-M_{1}}{M_{0}}*100\%$$

In the formula, P represents the loss rate (%); M_0_ is the initial amount of particle activated carbon added (g); M_1_ is the mass of particle activated carbon after loss (g).

#### 2.4.3 Determination of AC recovery rate

Thirty grams of organic fertilizer were weighed and placed in a 100 mL polyethylene bottle. Ultra-pure water was added to adjust the moisture content (55, 60, 65, 70%). 3 g of particle carbon was added, and the mixture was mixed evenly. Then it was placed on a flip-over shaker. After 7 days, the particle activated carbon and the organic fertilizer were separated. The particle activated carbon was rinsed clean and dried at 70 °C until a constant weight was achieved, and the weight was measured. All the experiments are in triplicate. The calculation of the recovery rate is as follows:


$$R=\frac{M_{1}}{M_{0}}*100\%$$

In the formula, R represents the recovery rate (%); M_0_ is the initial amount of added particle activated carbon (g); M_1_ is the mass of particle activated carbon after recovery (g).

#### 2.4.4 Compression strength test of AC

The compression strength of particle activated carbon is measured using an electronic universal testing machine. During the test, the loading rate is 0.2 mm/min, and the compressive stress endured by particle activated carbon during crushing is used to represent the compression strength of particle activated carbon.

### 2.5 Test indicators and test methods

After sampling, the organic fertilizer and activated carbon were separated. One part of the samples was naturally air-dried and then ground and sieved to determine their physical and chemical properties (pH, EC, CEC, organic carbon content) and changes in heavy metal content (total content, bioavailable content, content of each existing form). Another part of the samples was stored at −20 °C for the determination of antibiotic content.

The pH and electrical conductivity (EC) were measured by distillation water extraction, with a solid–liquid mass ratio of 1:5. The determination of organic carbon was carried out using the potassium dichromate oxidation - spectrophotometer method (organic carbon content × 1.724 = organic matter content). The cation exchange capacity was determined by the hexamminecobalt trichloride solution-spectrophotometric method; the total heavy metal content was determined by the four-acid digestion method of HCl-HNO_3_-HF-HClO_4_; the bioavailable form of heavy metal was determined by the diethylenetriamine pentaacetic acid - calcium chloride - triethanolamine (DTPA-CaCl_2_-TEA) extraction; the classification of heavy metal forms was determined by the improved BCR continuous extraction method. The concentrations of Cu and Zn were determined using a flame atomic absorption spectrometer. The extraction and determination of tetracycline hydrochloride and ciprofloxacin were, respectively, referred to “Determination of oxytetracyline, tetracyline, chlortetracycline and doxycycline content for organic fertilizers-HPLC method” and “Simultaneous determination of tetracyclines, fluoroquinolones, sulfonamides, macrolides and chloramphenicols in soil by HPLC method.”

## 3 Results and discussion

### 3.1 AC performance

#### 3.1.1 Compressive strength of AC

**Figure 2:**
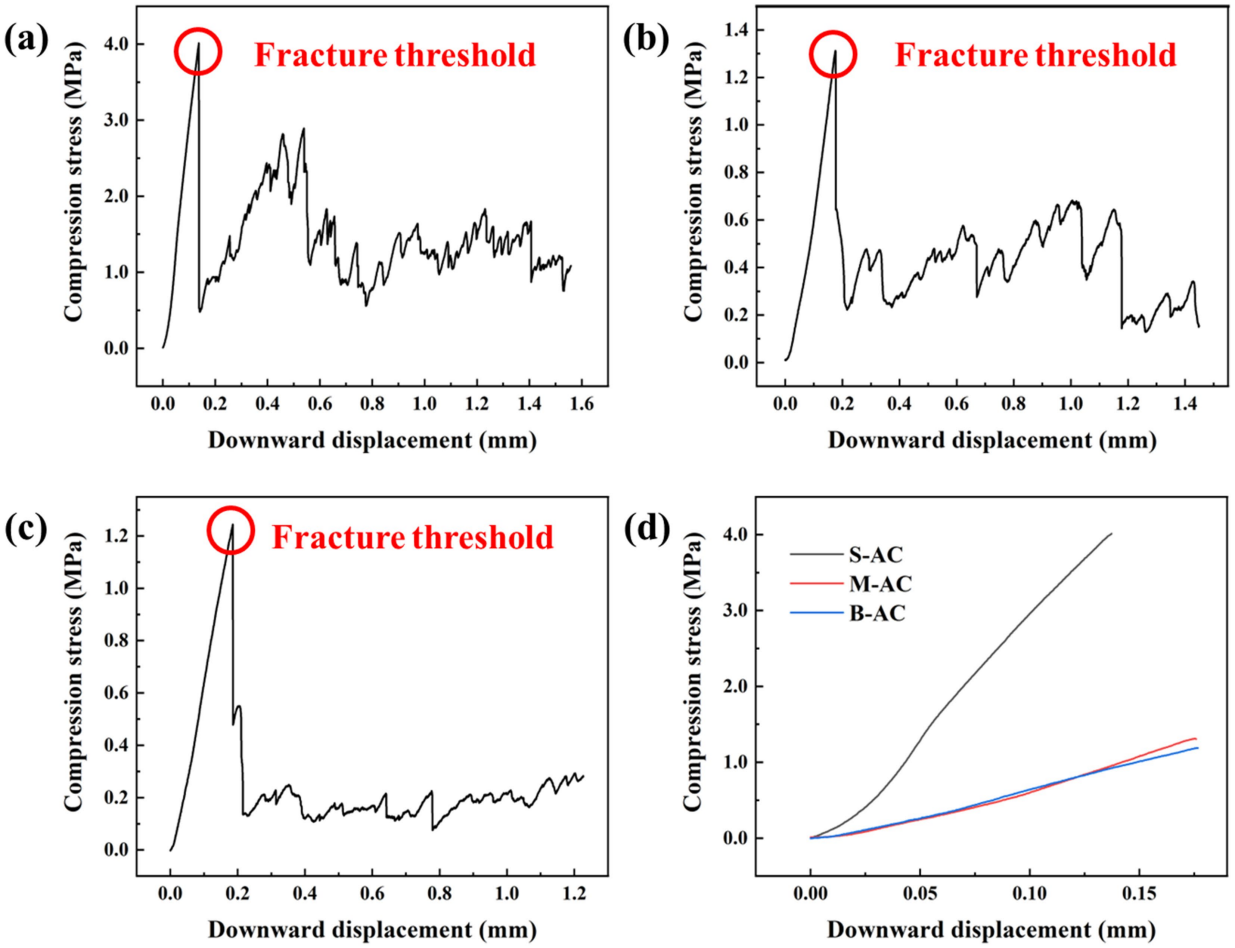
Compression performance of activated carbon: **(a)** S-AC; **(b)** M-AC; **(c)** B-AC; **(d)** Comparison of maximum compressive stress among different AC types.

The excellent compressive strength makes activated carbon more stable, preventing its fragmentation and the release of unstable components, which is beneficial for the separation of activated carbon from organic fertilizer and improving the recovery rate of activated carbon. This is of great significance for its practical application. Figure 2 shows the comparison results of the compressive performance of three different particle sizes of activated carbon. From the figure, it can be seen that in the initial stage, as the downward displacement increases, the compressive stress of the three types of particle activated carbon continues to rise, indicating that they exhibit certain elastic deformation ability when subjected to external pressure. However, when the pressure reaches the critical point, the particle activated carbon begins to fracture, and the compressive stress drops sharply. It is worth noting that the fragments of S-AC still have certain compressive strength after fracturing, indicating that S-AC can maintain a certain structural integrity even in the fractured state. In contrast, the fragmentation of medium and B-AC is more thorough, and the compressive strength of the fragments is much lower than that of S-AC, further highlighting the advantage of S-AC in mechanical strength.

To evaluate the compressive performance of the three types of particle activated carbon more objectively, the study takes the maximum compressive stress they can withstand as the evaluation index. The S-AC shows the most excellent compressive performance, with the compressive strength at the time of fracture reaching 4.014 MPa, which is significantly higher than that of M-AC (1.312 MPa) and B-AC (1.245 MPa). The reason for this phenomenon may be that compared with medium and large particle activated carbon, small particle activated carbon may have a more compact structural feature, fewer internal defects (such as cracks, pores), and fewer stress concentration points, resulting in a more uniform stress distribution when subjected to force, reducing the risk of fracture.

#### 3.1.2 Loss rate of AC

**Figure 3:**
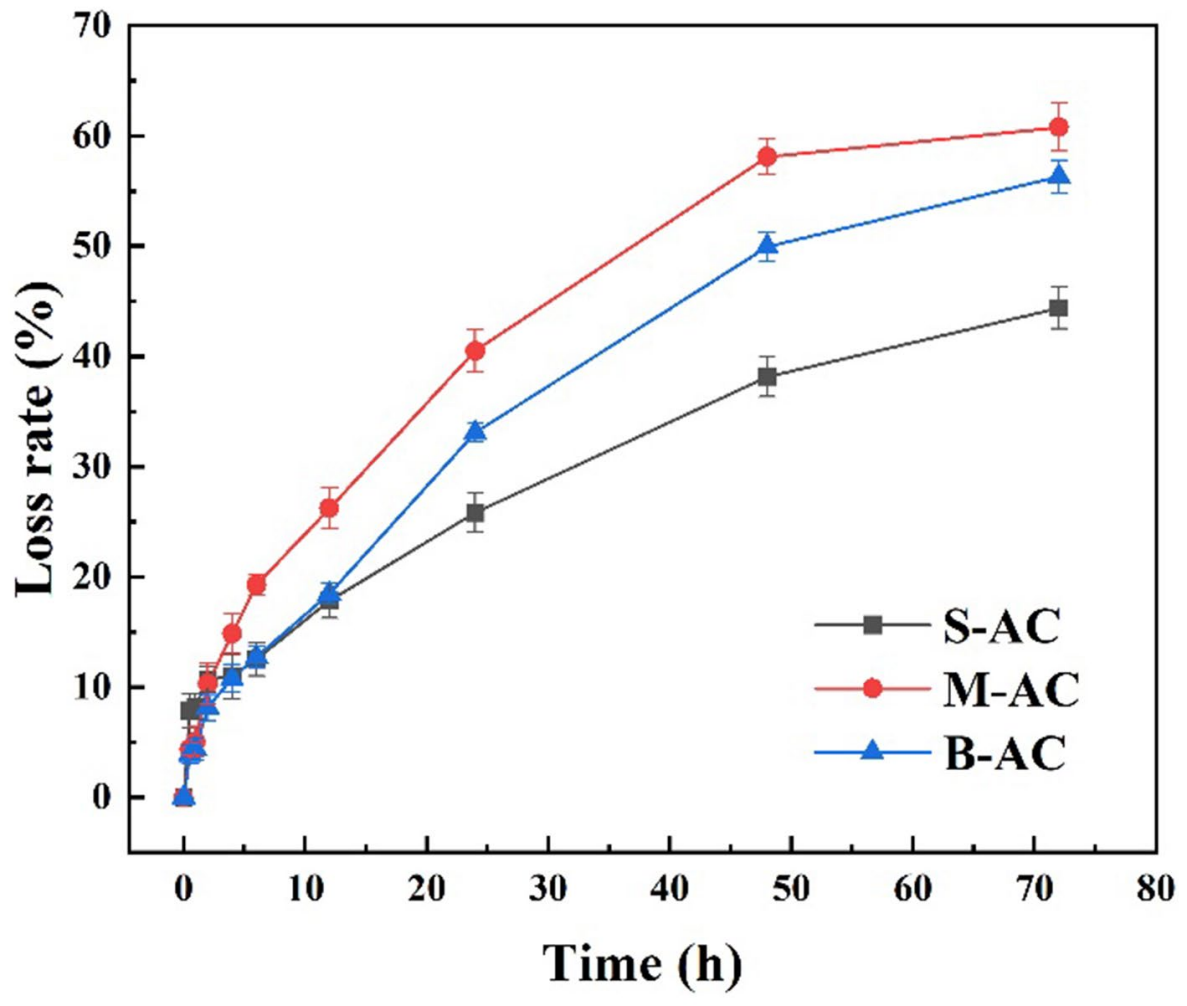
Evaluation of activated carbon loss rate.

The loss rate is one of the important indicators for assessing the mechanical strength of materials. Generally, the lower the loss rate, the higher the mechanical strength of the material and the stronger its anti-wear ability. Figure 3 shows the variation of loss rates of three different particle size particle activated carbon during the oscillation experiment. During the experiment, the particle activated carbon were subjected to continuous hydraulic flushing and mutual collisions between particles, resulting in a certain degree of wear on the surface and internal structure. With the extension of the oscillation time, the loss rates of the three particle activated carbon all showed a gradually increasing trend. After 72 h of oscillation experiment, the loss rates of S-AC, M-AC, and B-AC reached 44.39, 60.77, and 56.30%, respectively. The loss rate of S-AC was lower than that of M-AC and B-AC, which further verified that S-AC has higher mechanical strength. The low loss rate of S-AC is mainly attributed to its smaller particle size, which enables the carbon particles to have a closer contact and more effectively disperse external stress, thereby reducing wear and breakage.

#### 3.1.3 Recovery rate of AC

The moisture content is one of the key factors affecting the metal immobilization effect in organic fertilizers. An appropriate moisture content not only promotes the growth and metabolic activities of microorganisms but also enhances the adsorption and immobilization capacity of particle activated carbon for pollutants, thereby effectively reducing the mobility and bioavailability of pollutants in organic fertilizers. However, excessive moisture content will lead to a significant increase in the loss rate of particle activated carbon, affecting its recovery rate and remediation effect.

**Figure 4:**
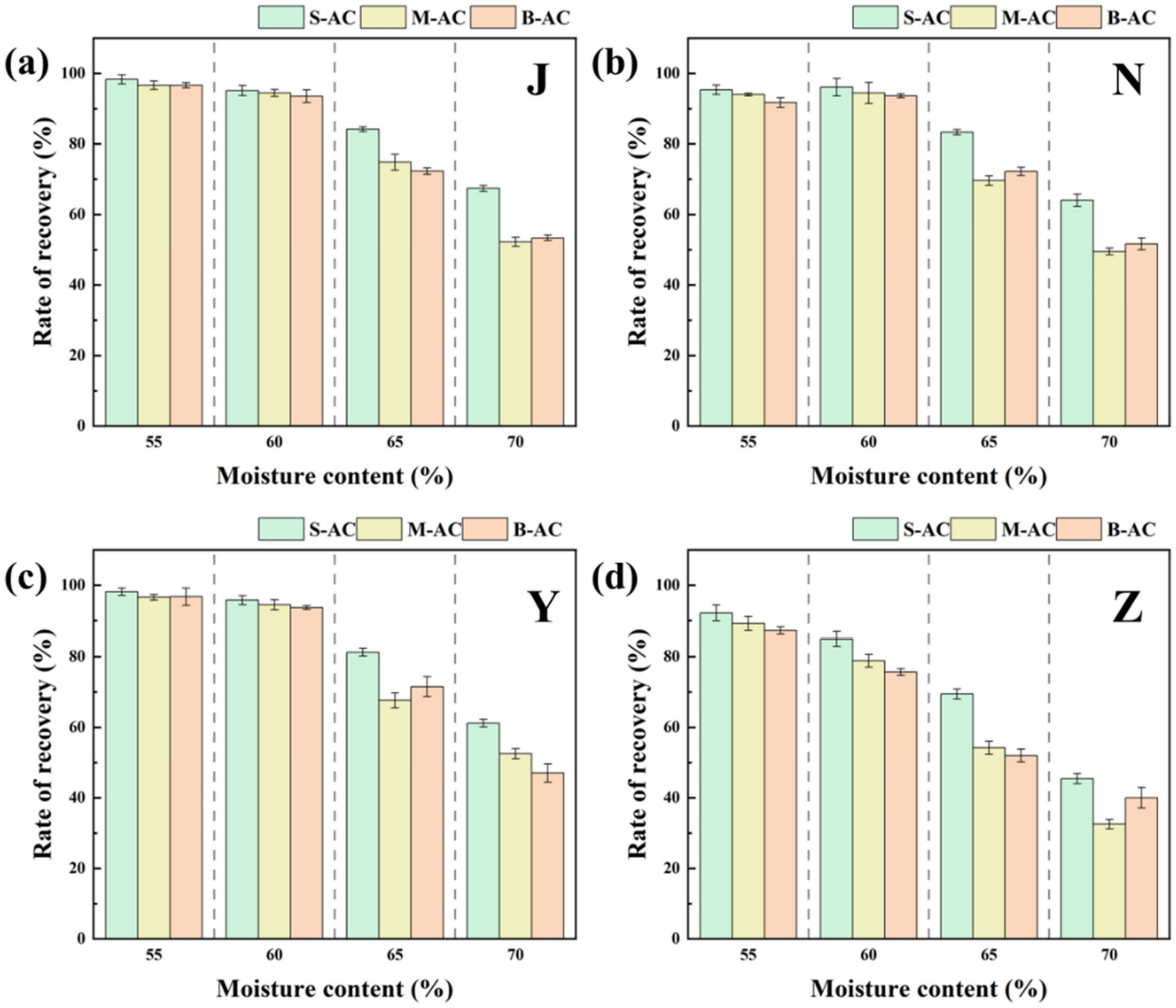
Recovery rate of AC under different moisture contents: **(a)** J group; **(b)** N group; **(c)** Y group; **(d)** Z group.

Figure 4 shows the recovery of particle activated carbon in four types of organic fertilizers under different moisture contents. The experimental results indicate that in the cases of 55 and 60% moisture content, the recovery rate of particle activated carbon in the chicken manure group, the cow manure group, and the sheep manure group all reached over 91.71%, demonstrating high stability. However, when the moisture content was further increased, the recovery rate of particle activated carbon in these three groups significantly decreased. The reason for this phenomenon lies in that when the moisture content is low, the organic fertilizer is in a slurry state, which can provide certain protection to particle activated carbon during the shaking process, reducing the possibility of its fragmentation; while when the moisture content is too high, the fluidity of the organic fertilizer increases, reducing the protective effect on particle activated carbon, and the flowing organic fertilizer will continuously wash the surface of particle activated carbon, accelerating its fragmentation. Under the same moisture content conditions, the fluidity of pig manure fertilizer is higher than that of the other three groups, so its recovery rate of particle activated carbon is lower than that of the other three groups. In the subsequent experiments, the moisture content was set at 60% to balance the adsorption performance and recovery rate of particle activated carbon.

### 3.2 Properties changes of organic fertilizers after AC remediation

#### 3.2.1 pH changes

**Figure 5:**
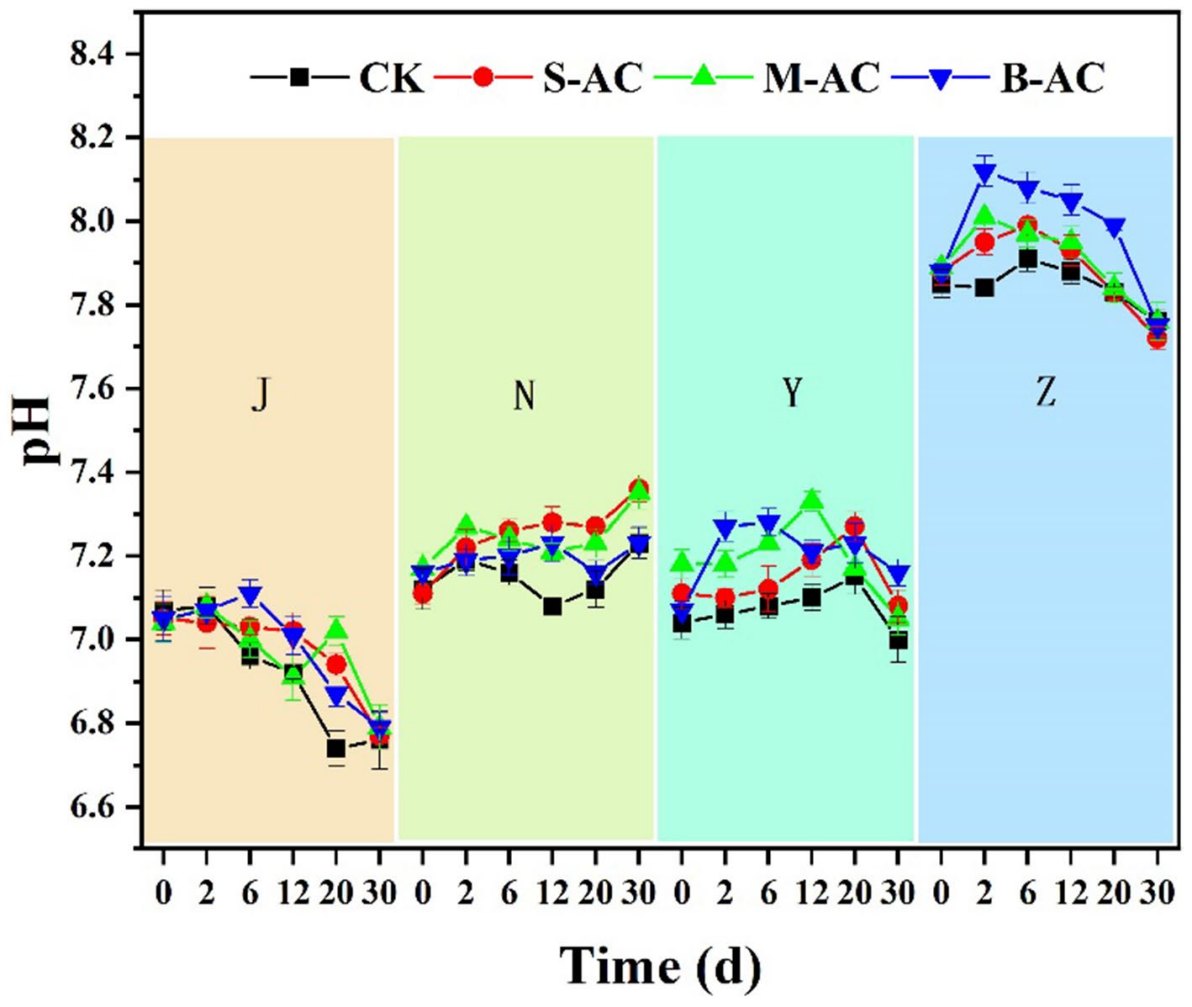
The variation of pH.

The dynamic changes in pH of each group of organic fertilizers are shown in Figure 5. The pH of the chicken manure group, cow manure group, and sheep manure group was neutral, while the pH of the pig manure group was slightly alkaline. Microorganisms thrive in neutral or slightly alkaline conditions, and a pH value within the range of 7–9 does not pose any harm to the life activities of microorganisms and is conducive to the process of aerobic composting. The pH of the chicken manure group showed a downward trend, with J-CK group, J-S group, J-M group, and J-B group decreasing by 0.31, 0.28, 0.25, and 0.26 units, respectively, at 30 days. The decrease in pH value might be due to the action of microorganisms, where organic matter in the organic fertilizer was decomposed, generating a large amount of small molecule organic acids (Onwosi et al., 2017; Wang et al., 2016). The pH of the cow manure group showed an upward trend, but the increase was not significant. At 30 days, N-CK group, N-S group, N-M group, and N-B group increased by 0.11, 0.25, 0.18, and 0.07 units, respectively. The increase in pH value might be due to the mineralization and decomposition of small molecule organic acids in the organic manure under the action of microorganisms. The pH of the sheep manure group and pig manure group increased first and then decreased. The difference was that the pH of the sheep manure group reached the peak at 12 days/20 days, and that of the pig manure group reached the peak at 2 days/6 days. At 30 days, except for Y-M group (an increase of 0.09 units), the pH of the other groups decreased slightly compared to the initial moment.

The increase and decrease of pH value first and then second might be due to the metabolic activities of microorganisms in the initial stage, where organic matter was decomposed to produce ammonia gas, causing an increase in pH. Subsequently, a large amount of organic matter was decomposed into small molecule organic acids, causing a decrease in pH. It is worth noting that the pH values of the S-AC group, M-AC group, and B-AC group of the four organic fertilizers were higher than that of the CK group. This might be because the pH values of the three types of activated carbon were higher than those of the organic fertilizers, and during the restoration process, some of the activated carbon cracked, resulting in an increase in the pH of the organic fertilizer; in addition, after applying the activated carbon, the alkaline functional groups and carbonates in the activated carbon could be released, leading to an increase in the pH value of the organic fertilizer. Secondly, the strong adsorption capacity of activated carbon could adsorb acidic substances (H^+^ and organic acids) in the organic fertilizer, reducing the acidic components in the organic fertilizer, thereby slowing down the acidification of the organic fertilizer and increasing the pH.

#### 3.2.2 EC changes

**Figure 6:**
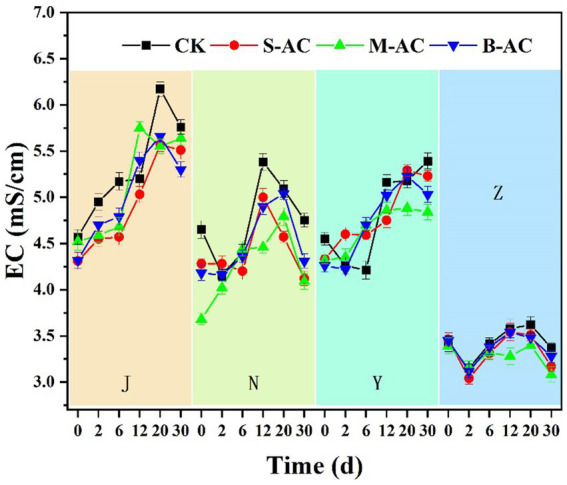
The variation of EC.

Electrical conductivity (EC) can be used to indicate the content of soluble salts in organic fertilizers. If the EC value is too high, it will cause salt damage to crops, which is not conducive to their growth; if it is too low, it will lead to insufficient nutrition for the crops. The dynamic changes of EC values in each experimental group are shown in Figure 6. Except for the pig manure group, the electrical conductivities of the other three organic fertilizers were all higher than the EC value (4 mS/cm) that limits crop growth. The EC values of the chicken manure group and the cow manure group first increased and then decreased. The difference is that the EC value of the 30-day chicken manure group (5.30–5.76) was significantly higher than the initial EC value (4.31–4.57), while the EC value of the cow manure group (4.09–4.75) basically returned to the initial EC value (3.68–4.65); the EC value of the sheep manure group showed an upward trend, and the EC value of the 30-day group (4.84–5.39) was significantly higher than the initial EC value (4.26–4.55).

The increase in EC values in the early stage may be due to the decomposition of organic materials by microorganisms in the organic fertilizers and the generation of a large amount of small molecule organic acids, HCO_3_^−^, HSO_4_^−^, H^+^, NH_4_^+^, and phosphate salts, etc (Temel, 2023); the decrease in EC values in the later stage may be due to the reduction in the content of easily degradable organic matter and the large volatilization of gas substances such as CO_2_ and NH_3_, which led to a decrease in the content of soluble ions in the organic fertilizers (Ghinea and Leahu, 2020). The EC value of the pig manure group first decreased, then increased, and then decreased again. The reason for the decrease in EC values in the initial stage may be the volatilization of ammonium nitrogen and the degradation of small molecule organic acids. The changes in the electrical conductivities of the three particle carbon groups were not significantly different from those of the control group, indicating that the addition of particle carbon had no significant effect on the changes in electrical conductivity.

#### 3.2.3 CEC changes

**Figure 7:**
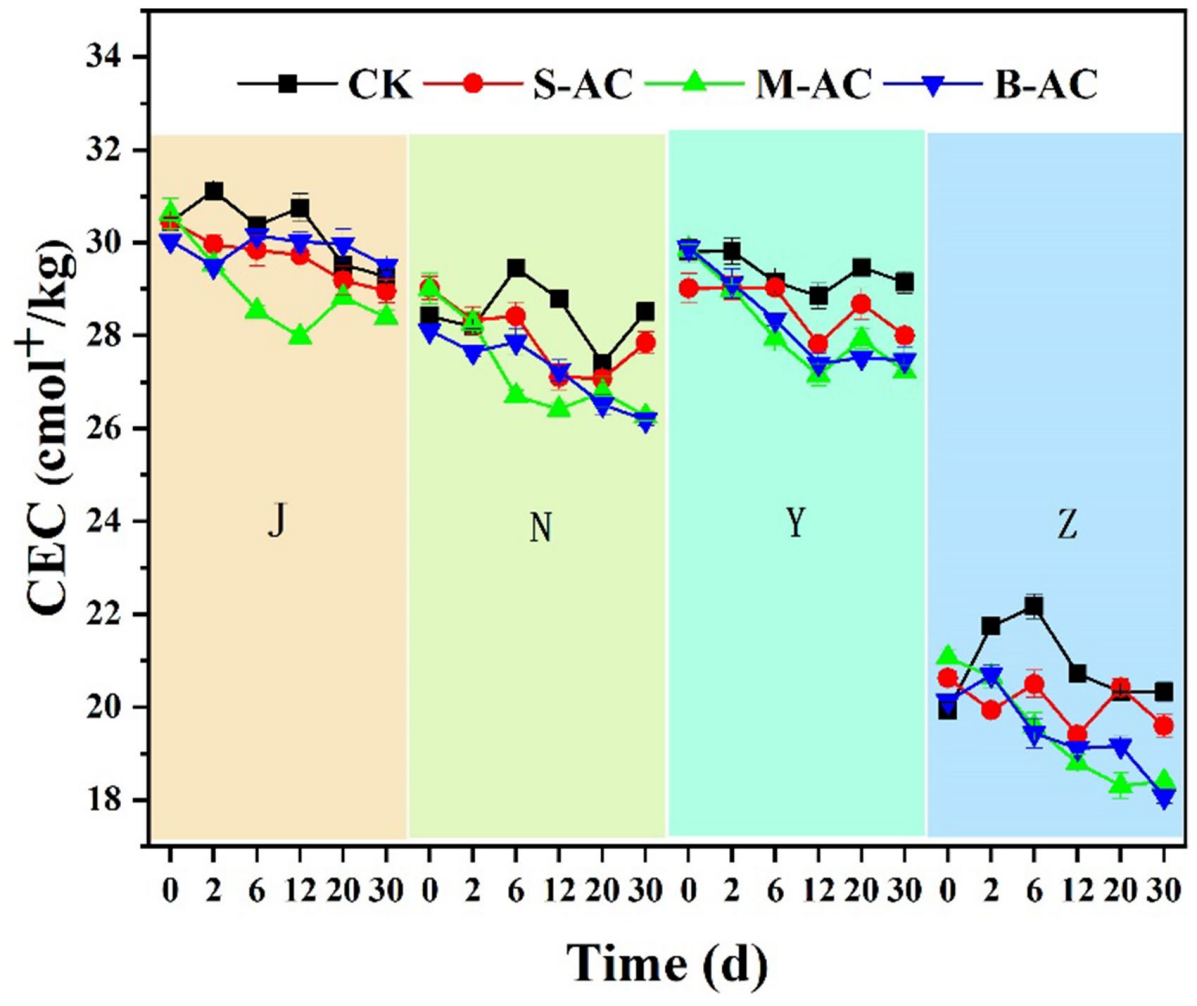
The variation of CEC.

CEC refers to the total amount of various cations that can be adsorbed in organic fertilizers, which affects the transformation of substances and the transport of solutes in the fertilizers, and can reflect the fertility and buffering capacity of the organic fertilizers. Figure 7 shows the changes in CEC over time after adding particles of different particle sizes of activated carbon to contaminated organic fertilizers. As shown in the figure, except for the N-CK and Z-CK groups, the CEC values of the other experimental groups showed a decreasing trend. After 30 days of remediation, the CEC of the J-CK, J-S, J-M, and J-B groups decreased by 1.18, 1.51, 2.23, and 0.54 cmol^+^/kg respectively; the CEC of the N-S, N-M, and N-B groups decreased by 1.17, 2.74, and 1.92 cmol^+^/kg (the CEC of the N-CK group increased by 0.10 cmol^+^/kg); the CEC of the Y-CK, Y-S, Y-M, and Y-B groups decreased by 0.66, 1.02, 2.62, and 2.41 cmol^+^/kg respectively; the CEC of the Z-S, Z-M, and Z-B groups decreased by 1.03, 2.66, and 2.06 cmol^+^/kg (the CEC of the Z-CK group increased by 0.40 cmol^+^/kg).

The treatment groups with activated carbon addition had a greater decrease in CEC compared to the control group. This may be because activated carbon adsorbed a large amount of heavy metal ions during the remediation process. As the activated carbon separated from the organic fertilizer, these adsorbed ions were also carried away, resulting in a decrease in the exchangeable cations in the organic fertilizer and a decrease in CEC. The organic matter in the organic fertilizer contains a large number of functional groups such as carboxyl and hydroxyl groups, which can adsorb and exchange cations, thereby increasing the CEC value of the organic fertilizer; in addition, the large specific surface area of the organic matter can provide more adsorption sites, which can further enhance the CEC value of the organic fertilizer. The adsorption of some organic matter in the organic fertilizer by activated carbon and its removal from the organic fertilizer is also the reason for the decrease in CEC.

#### 3.2.4 Organic matter changes

**Figure 8:**
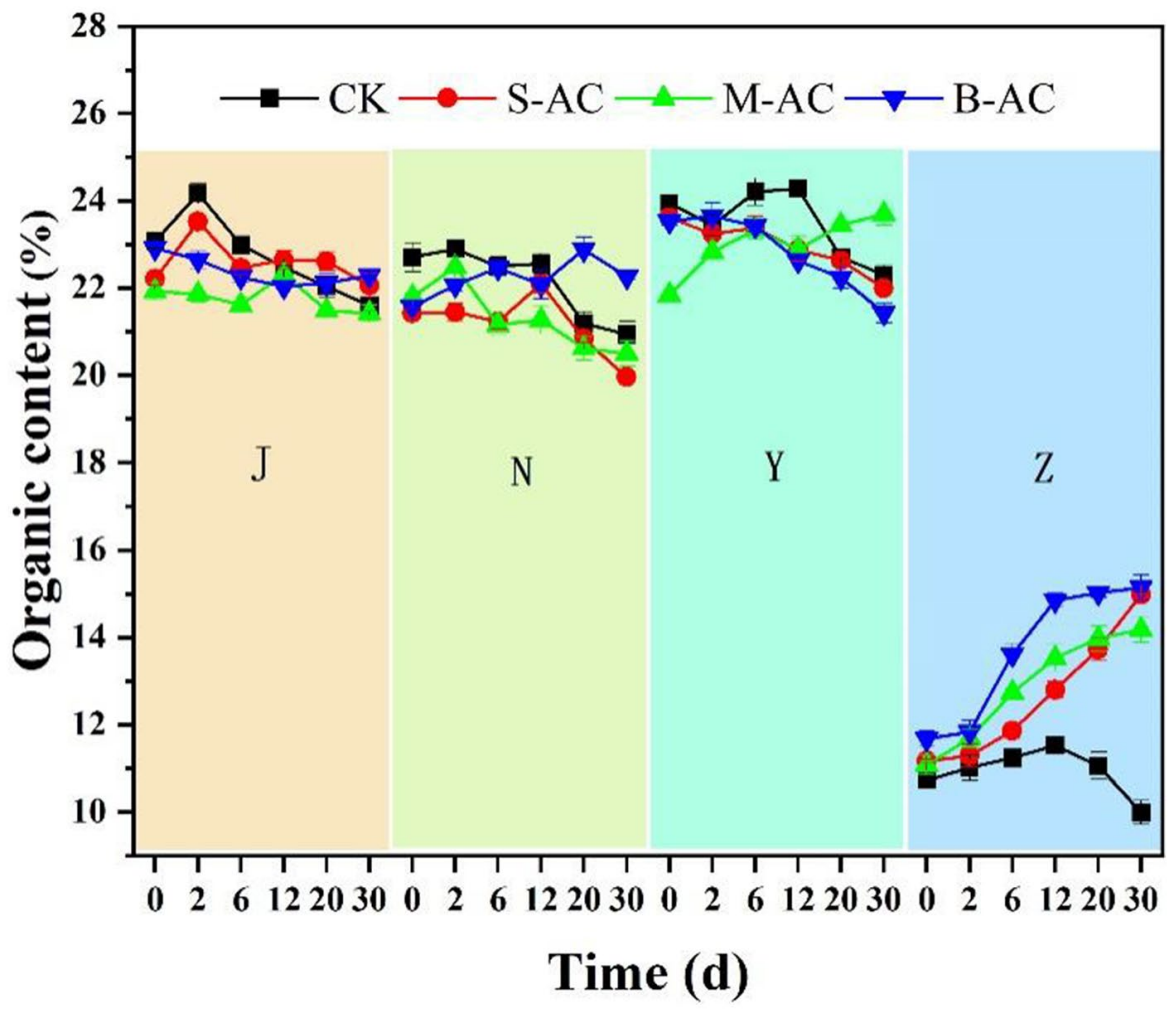
The variation of organic content.

The changes in organic matter content of the four groups of organic fertilizers with different particle sizes are shown in Figure 8. Under the action of microorganisms, organic matter is consumed and converted into CO_2_ and humus substances (Zhao et al., 2023; Yang et al., 2022). Therefore, the organic matter content in the chicken manure group, cow manure group, and sheep manure group shows a downward trend. However, the N-B group, Y-M group, especially the Z-S, Z-M, and Z-B groups have significantly increased organic matter. The possible reason is that some particle carbons break during the shaking process and mix with the organic fertilizers, and the activated carbon itself has a high organic carbon content, resulting in an increase in the organic matter content of the organic fertilizers. The reasons for the decrease in organic matter content of each experimental group after applying activated carbon also include that activated carbon, due to its developed pore structure, can adsorb the organic matter in the organic fertilizers; the active groups on the surface of activated carbon (such as hydroxyl, carboxyl, etc.) can also form stable compounds with the organic matter, and when the activated carbon is separated, the adsorbed organic matter and the formed compounds are removed, resulting in a decrease in the organic matter content.

### 3.3 Heavy metal passivation

#### 3.3.1 Changes in total content and occurrence forms

**Figure 9:**
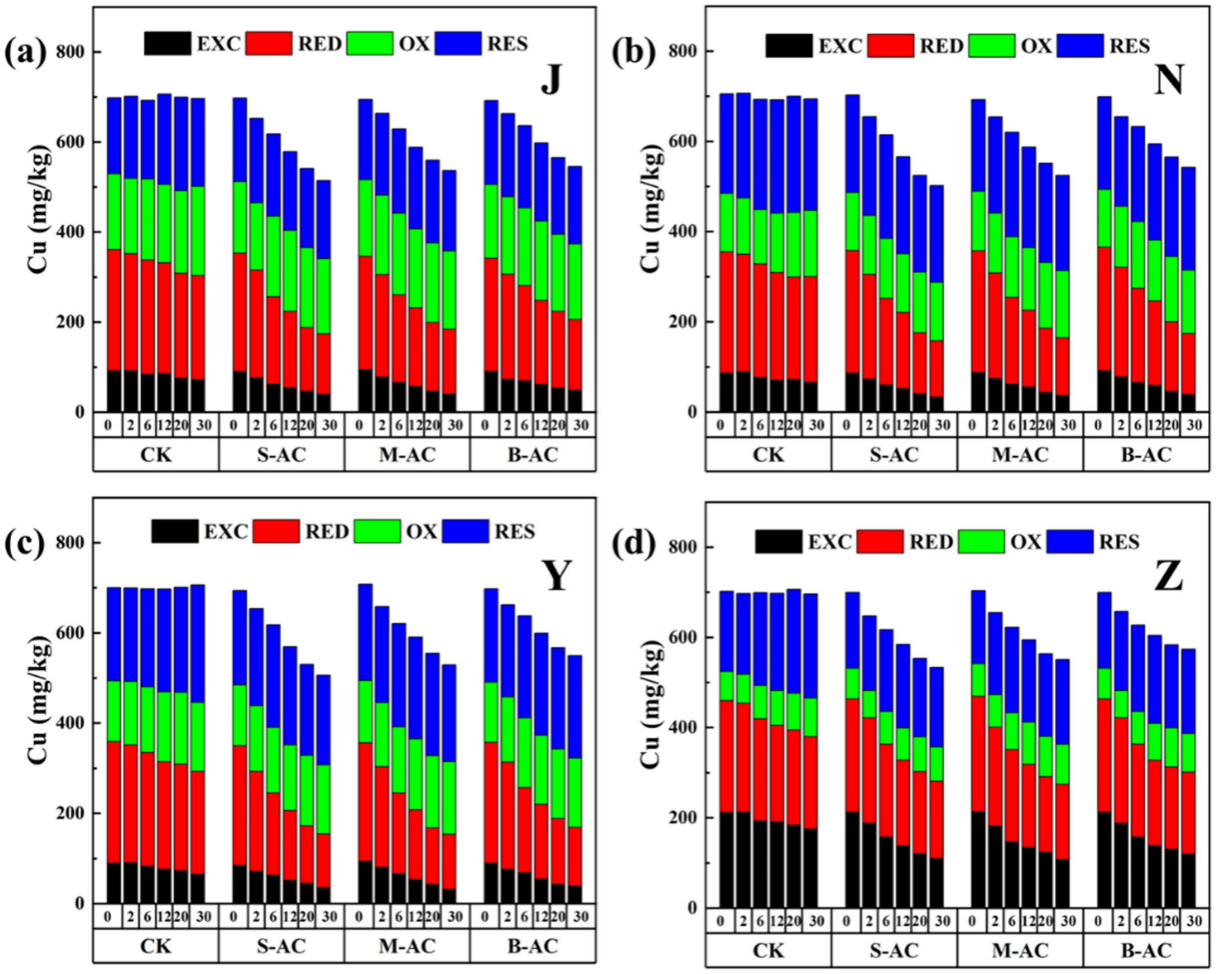
Changes in total Cu content and various forms of Cu: **(a)** J group; **(b)** N group; **(c)** Y group; **(d)** Z group.

**Figure 10:**
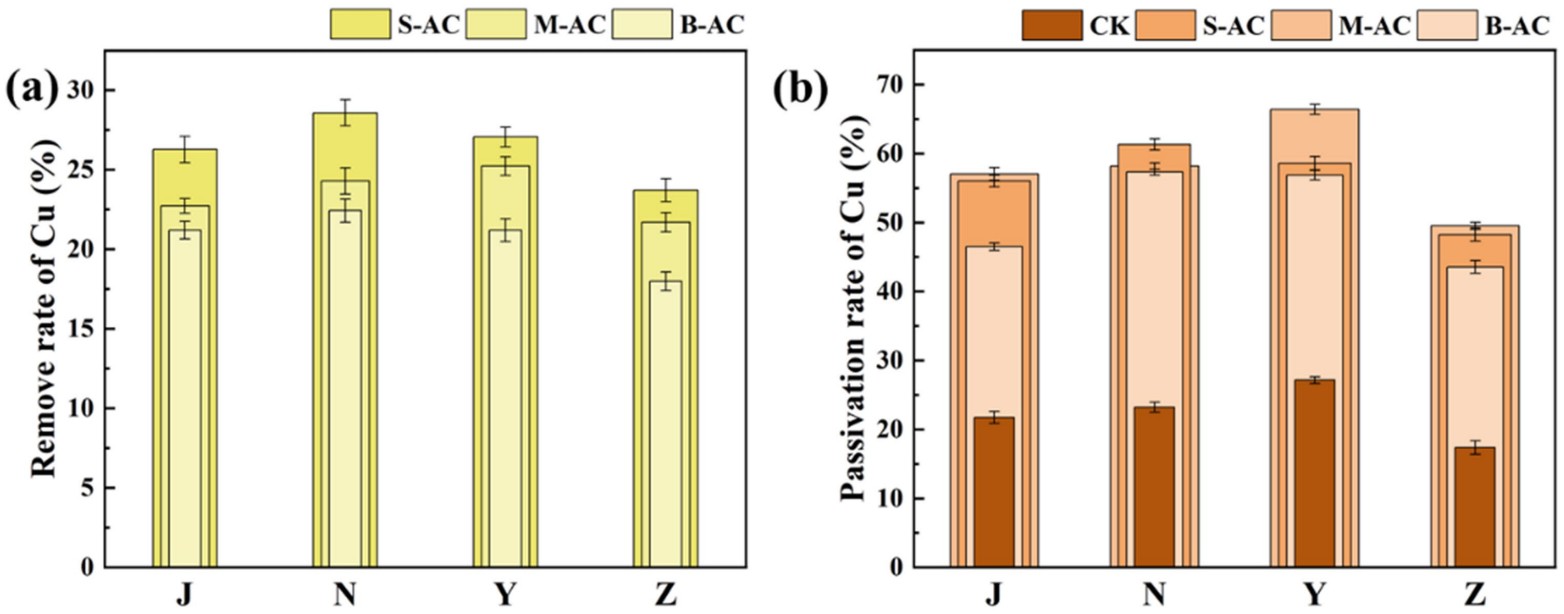
**(a)** Total Cu removal rate; **(b)** Cu passivation rate of exchangeable fraction.

Figure 9 shows the total content of Cu in the organic fertilizer before and after the addition of particle activated carbon, as well as the changes in each occurrence form. It can be clearly seen from the figure that after particle activated carbon was added to the organic fertilizer, the total content of Cu gradually decreased over time. This result indicates that particle activated carbon has a significant adsorption and fixation effect on Cu, and can effectively reduce the content of Cu in the organic fertilizer. Figure 10a further shows the removal effect of Cu total content in each group of organic fertilizers at 30 days. The S-AC groups of all four types of organic fertilizers had the best Cu removal effect. The copper total removal rates of the S-AC groups in the 30-day chicken manure fertilizer group, cow manure fertilizer group, sheep manure fertilizer group, and pig manure fertilizer group were 26.28, 28.57, 27.05, and 23.71%, respectively. The S-AC group and the B-AC group had the best Cu removal effect in the cow manure fertilizer group; the M-AC group had the best Cu removal effect in the sheep manure fertilizer group. The particle activated carbon is alkaline and, to a certain extent, increases the pH value of the organic fertilizer by adding it, which can convert heavy metal ions into hydroxides and form precipitates adhering to the surface of the particle carbon (Cui et al., 2019; Moreno-Barriga et al., 2017). In addition, the particle carbon has a large static electric force and cavity surface, and the various groups on the surface react with the heavy metal ions in the organic fertilizer through coordination and ion exchange reactions, adsorbing heavy metals (Liu et al., 2020; Lin et al., 2019).

The toxicity of heavy metals in organic fertilizers is not only closely related to their total amount, but also affected by the distribution of their chemical forms. The chemical forms of heavy metals determine their mobility and toxicity, and changes in forms can significantly affect their behavior in the environment (Lu et al., 2017; Nomeda et al., 2008). The activity of the four existing forms of heavy metal is from high to low as follows: exchangeable fraction EXC (adsorbed by electrostatically on the surface of organic fertilizer particles, an element form that can be released by ion exchange, and the element form bound to carbonate), reducible fraction RED (element form held by iron oxide, manganese oxide, etc.), oxidizable fraction OX (element form bound to active groups of organic matter, and the element form of sulfide oxidized to soluble sulfate form), and residual fraction RES (element form existing in the silicate lattice).

As shown in Figure 9, before and after the application of AC, Cu in the chicken manure group, cow manure group, and sheep manure group mainly exists in the reducible form, followed by the oxidizable fraction and the residual fraction, while the proportion of the exchangeable fraction is the lowest. Cu in the pig manure group also mainly exists in the reducible form, but is followed by the exchangeable fraction and the residual fraction, and the proportion of the oxidizable fraction is the lowest. Compared with the control CK group, the proportion of the exchangeable fraction and reducible fraction of Cu in the four organic fertilizers under different particle activated carbon treatments has decreased. This change indicates that the addition of particle activated carbon can effectively reduce the bioavailability and potential toxicity of Cu, thereby reducing its harm to the environment. The reduction of exchangeable fraction and reducible fraction has two reasons: (1) Adsorbed by particle activated carbon and separated from the organic fertilizer; (2) Converted to more stable forms (oxidizable fraction, residual fraction).

The exchangeable fraction of heavy metals has high plant availability and is easily utilized by plants. Therefore, in this study, the exchangeable fraction was used as the indicator to measure the metal passivation effect. Figure 10b shows the Cu passivation rates of each group of organic fertilizers. From the figure, it can be seen that adding activated carbon can significantly increase the Cu passivation rate. Specifically, in the chicken manure group, the sheep manure group, and the pig manure group, the passivation effect of particle activated carbon was the best, with Cu passivation rates reaching 57.04, 66.39, and 49.49%, respectively. In the cow manure group, the passivation effect of S-AC was the most significant, with a passivation rate of 61.31%. The passivation effect of Cu in the S-AC group and the B-AC group was the best for cow manure fertilizer; the passivation effect of Cu in the M-AC group and the sheep manure fertilizer was the best.

**Figure 11:**
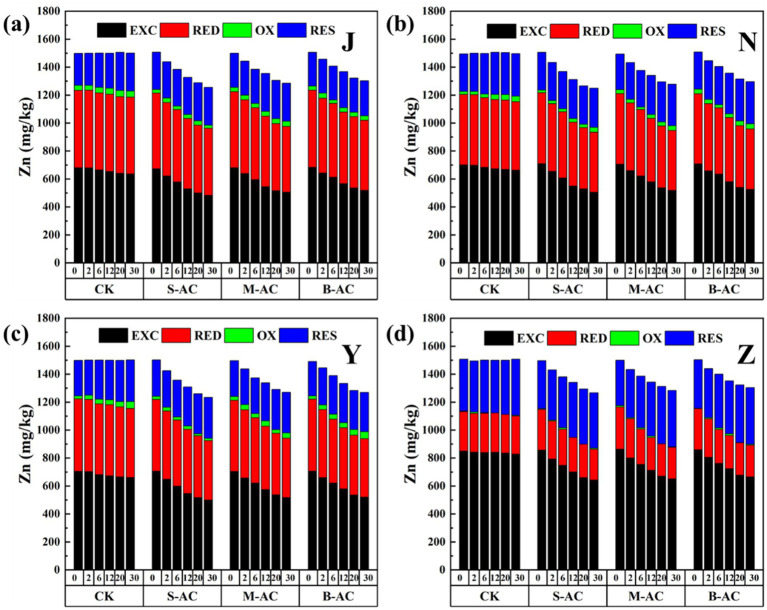
Changes in total Zn content and various forms of Zn: **(a)** J group; **(b)** N group; **(c)** Y group; **(d)** Z group.

**Figure 12:**
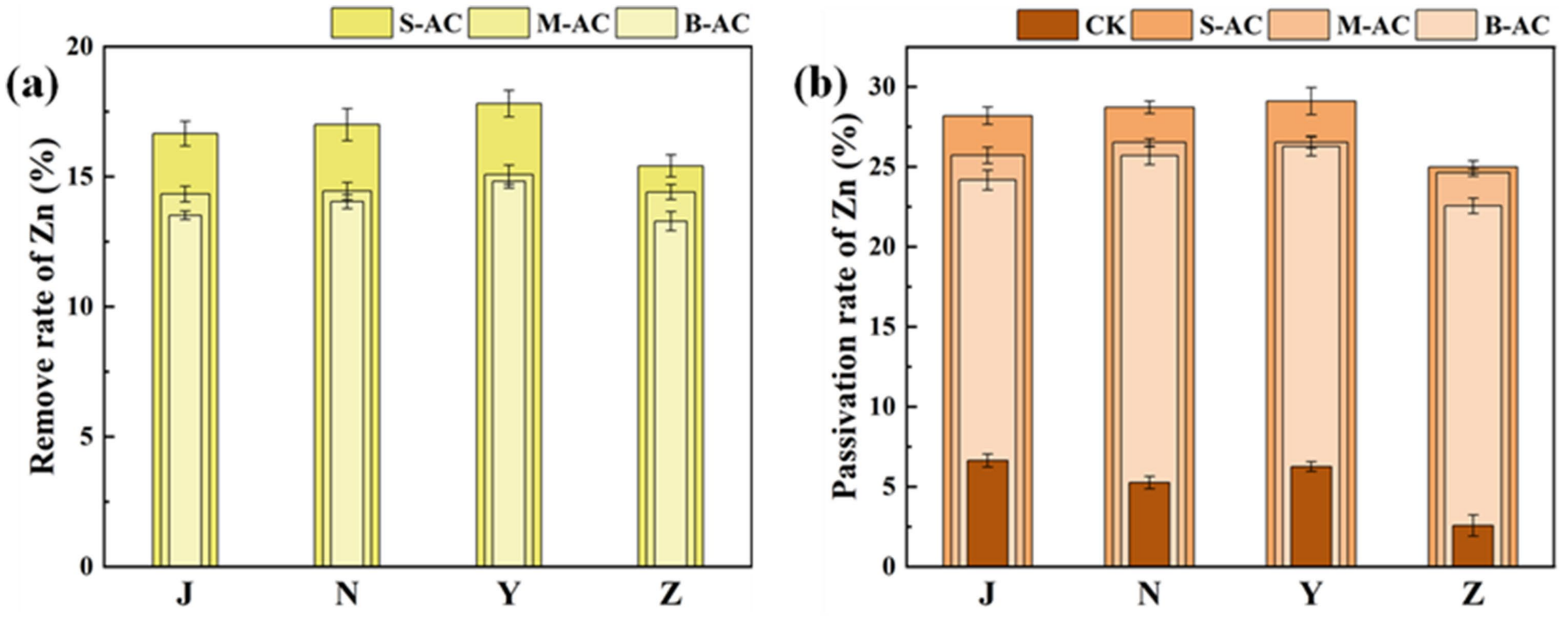
**(a)** Total Zn removal rate; **(b)** Zn passivation rate of exchangeable fraction.

Figures 11, 12 show the changes in the total Zn content and its distribution forms in the organic fertilizer after adding particles of different diameters. Similar to Cu, after the addition of particle carbon, the total Zn content gradually decreased. The removal effect of Zn by S-AC was the best, with the Zn total removal rates in the chicken manure group, cow manure group, sheep manure group, and pig manure group being 16.66, 17.00, 17.81, and 15.41%, respectively. All three groups of particle carbon had the best Zn removal effect for sheep manure fertilizer. Different from Cu, the Zn in the four groups of organic fertilizers mainly existed in the exchangeable fraction, followed by reducible fraction and residual fraction, and the oxidizable fraction was almost non-existent. Compared with the control (CK) group, different particle carbon treatments could reduce the content of exchangeable fraction of Zn in the organic fertilizer. The Zn passivation rates of the four groups of organic fertilizers were 28.19, 28.72, 29.10, and 24.99%, respectively. The Zn passivation effect of the S-AC group, B-AC group, and sheep manure fertilizer group was the best; the Zn passivation effect of the M-AC group and cow manure fertilizer group was the best.

The application of particle activated carbon not only effectively reduces the total Cu and Zn content in the organic fertilizer, but also changes the chemical form distribution of Cu and Zn, reducing their bioavailability and toxicity, thereby promoting the solidification and stabilization of heavy metals. This process helps to reduce the potential risks of heavy metals to the environment, improve the safety and usability of the organic fertilizer. The particle size of the activated carbon has a significant impact on the removal and passivation effect of Cu and Zn. The SAC, due to its larger specific surface area and rich pore structure, can more effectively adsorb and fix heavy metal ions, thus showing better removal and passivation effects.

#### 3.3.2 Changes in the bioavailable form of heavy metals

**Figure 13:**
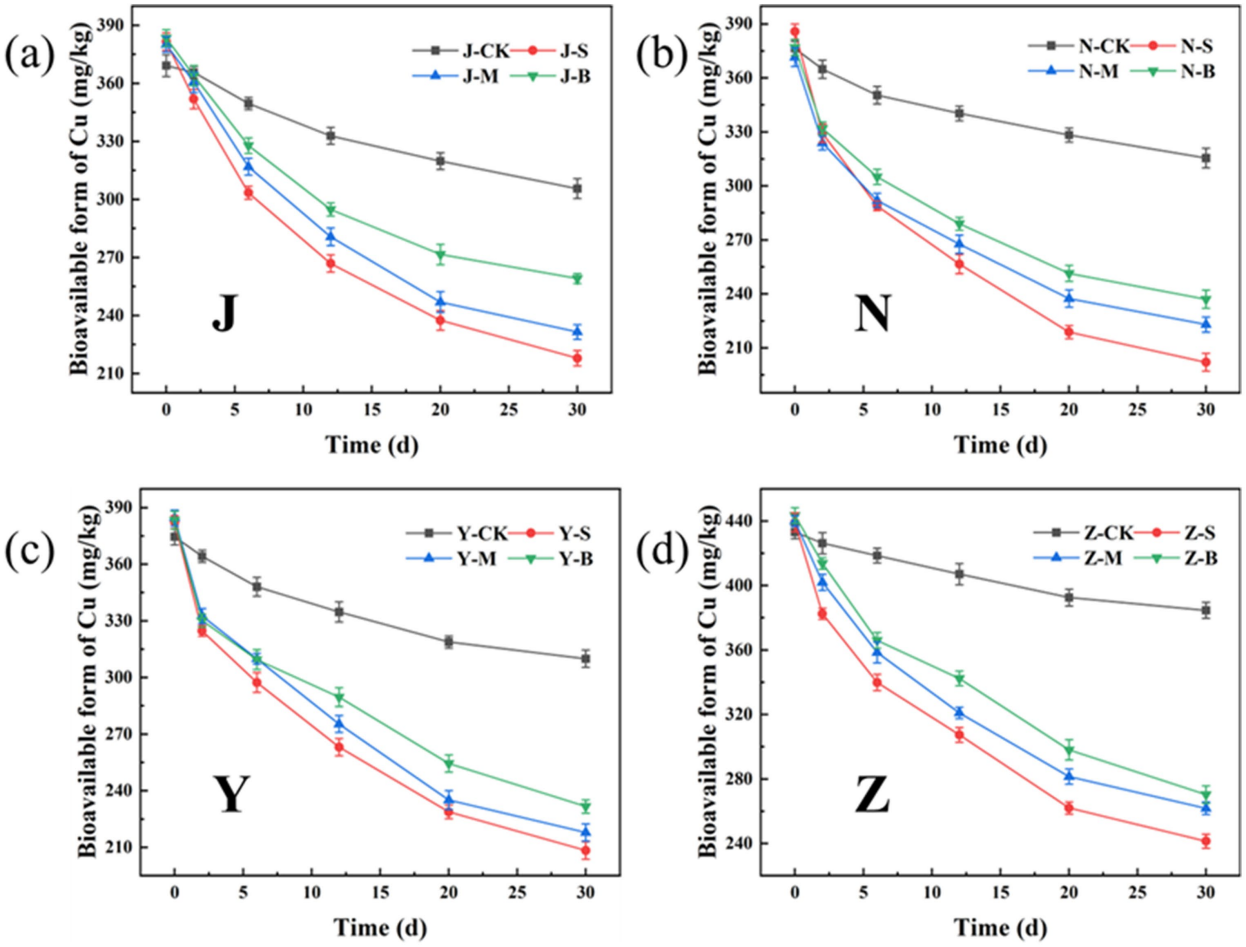
Changes in the bioavailable form of Cu: **(a)** J group; **(b)** N group; **(c)** Y group; **(d)** Z group.

**Figure 14:**
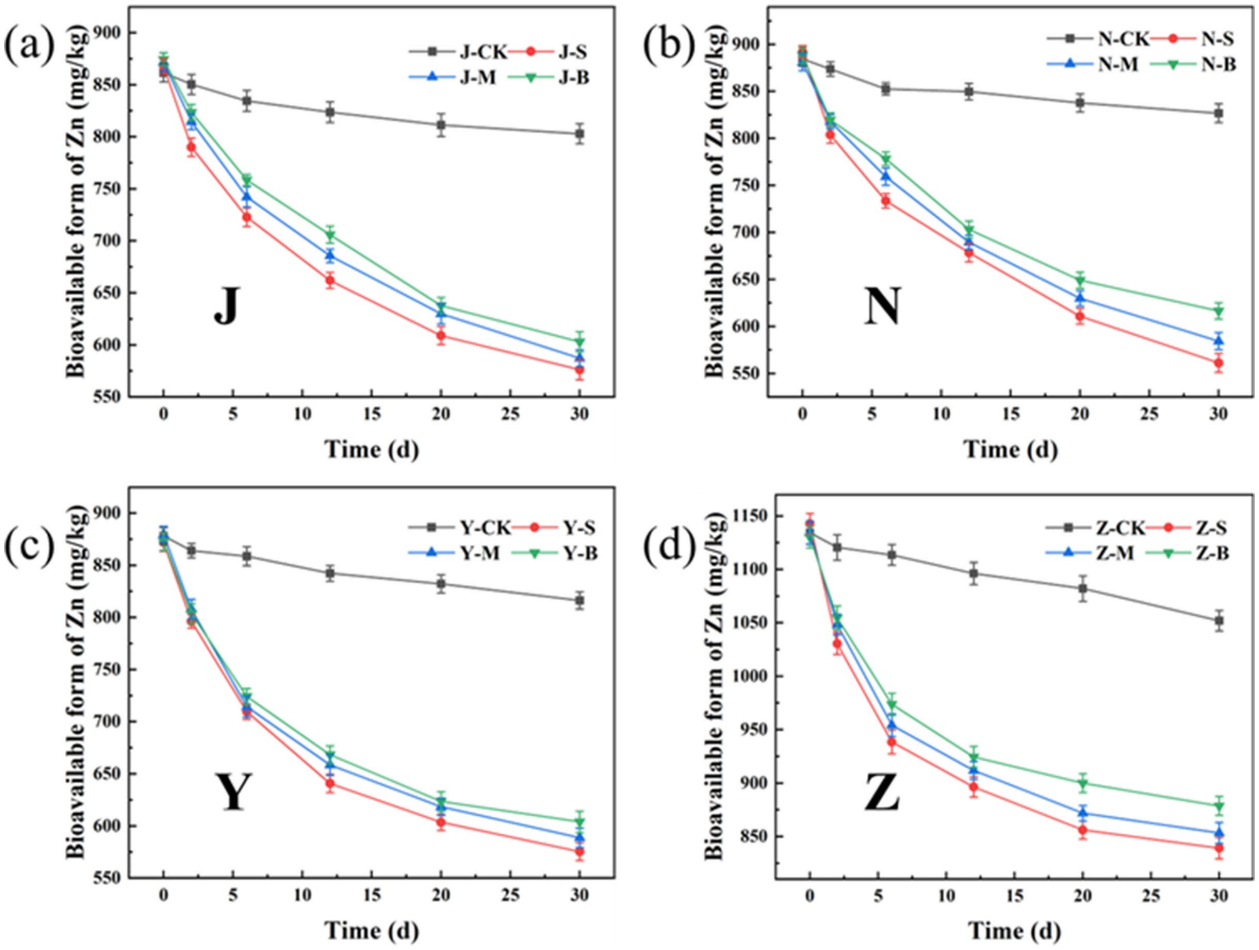
Changes in the bioavailable form of Zn: **(a)** J group; **(b)** N group; **(c)** Y group; **(d)** Z group.

**Figure 15:**
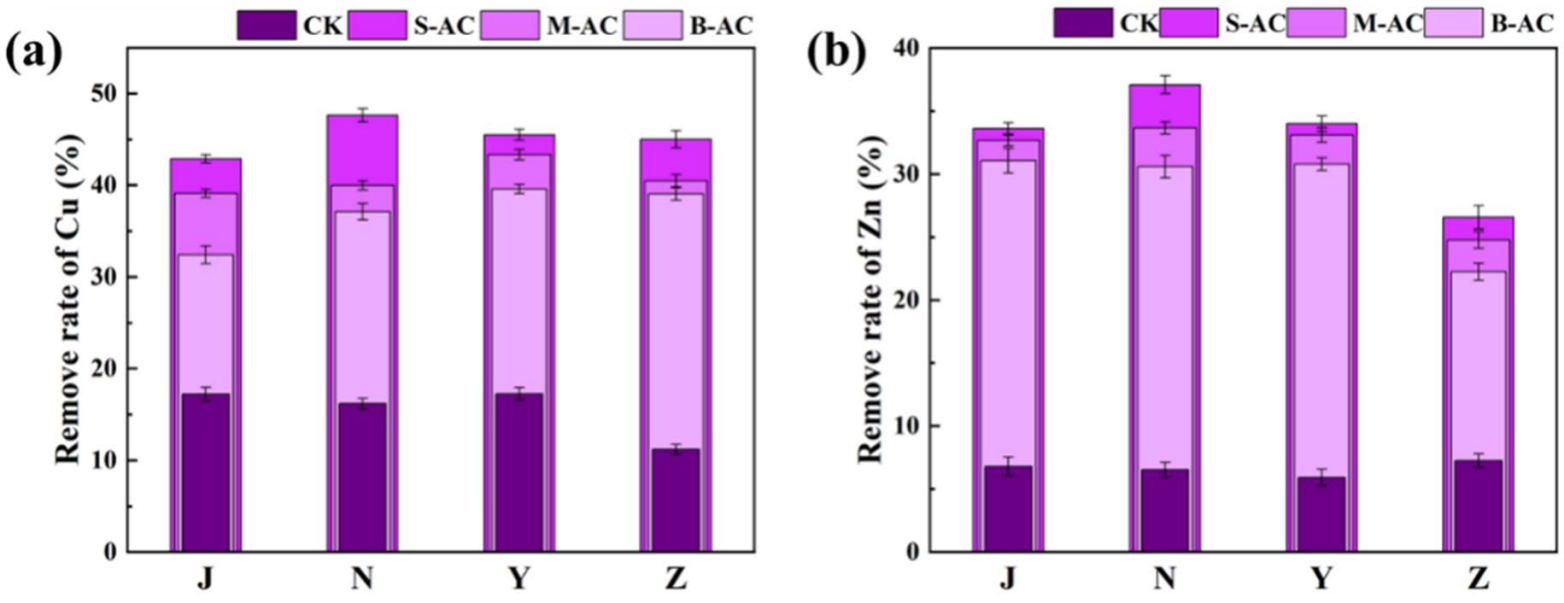
Removal rate of bioavailable form of heavy metals: **(a)** Cu; **(b)** Zn.

The bioavailable form of heavy metals refers to the form in which heavy metals can be absorbed and utilized by organisms, with high chemical activity and strong mobility. Figures 13, 14 show the changes in the bioavailable Cu and Zn in the organic fertilizer after the application of three particle sizes of activated carbon. Compared with the CK group, the bioavailable Cu and Zn significantly decreased after the application of carbon particles. The chelation effects of the organic fertilizers on the available Cu and Zn in all four groups were as follows: the small particle carbon had the best effect, followed by the medium particle carbon, and the large particle carbon had the worst effect. Figure 15 shows the removal rates of bioavailable Cu and Zn after 30 days. The removal rates of bioavailable Cu in the chicken manure group, cow manure group, sheep manure group, and pig manure group (S-AC) were 42.89, 47.64, 45.50, and 45.03%, respectively; the removal rates of bioavailable Zn (S-AC) were 33.61, 37.08, 34.00, and 26.58%, respectively. The removal rate of bioavailable Cu in the sheep manure fertilizer with S-AC was the best; the removal rates of bioavailable Cu and Zn in the M-AC and B-AC groups of sheep manure fertilizer were the best. The carbon fixed and chelated the forms of heavy metals Cu and Zn into a state with low toxicity and not easily transported to the plant body, playing a positive role in the stabilization and fixation of Cu and Zn.

### 3.4 Changes in antibiotic content

**Figure 16:**
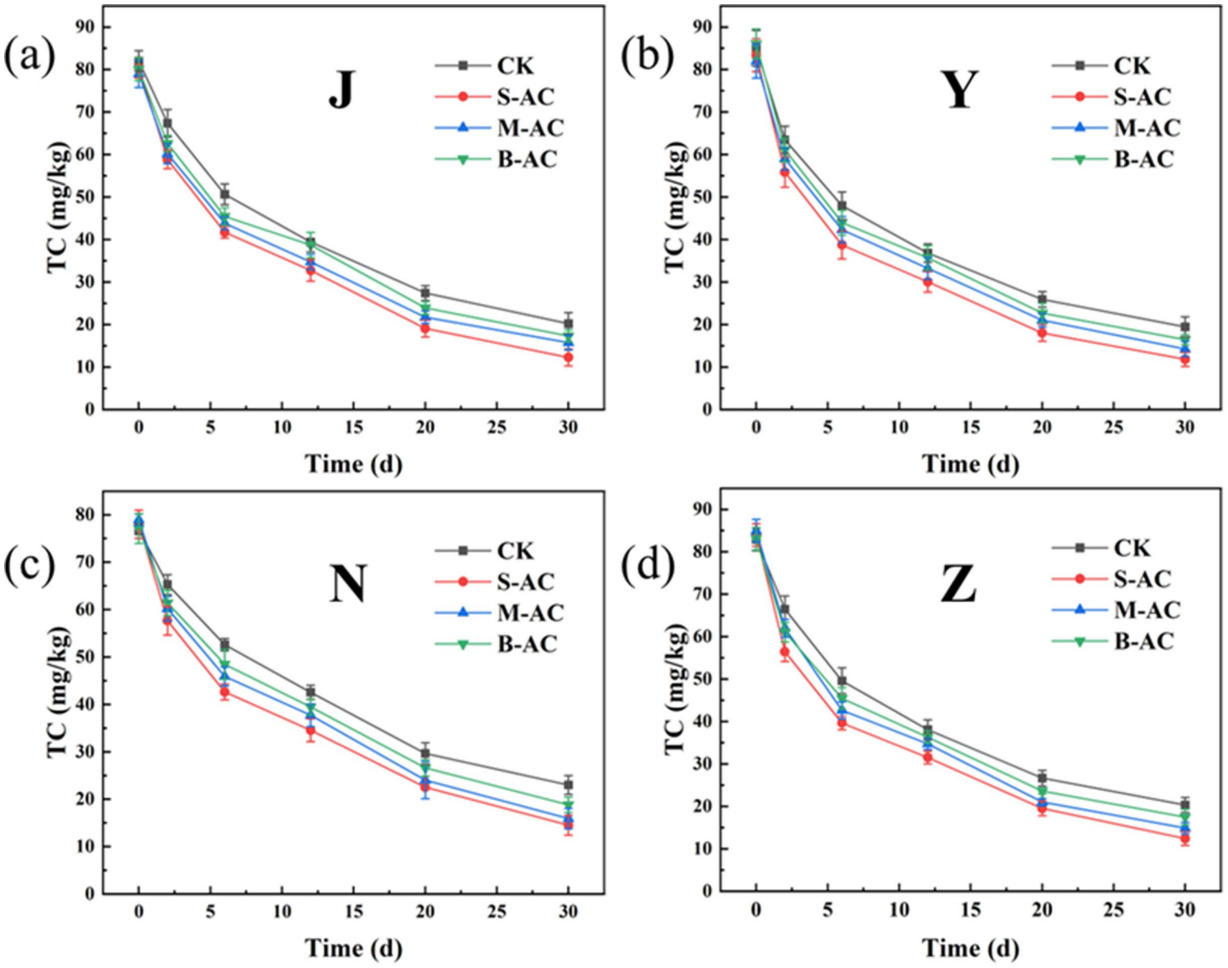
Changes in TC concentration: **(a)** J group; **(b)** Y group; **(c)** N group; **(d)** Z group.

**Figure 17:**
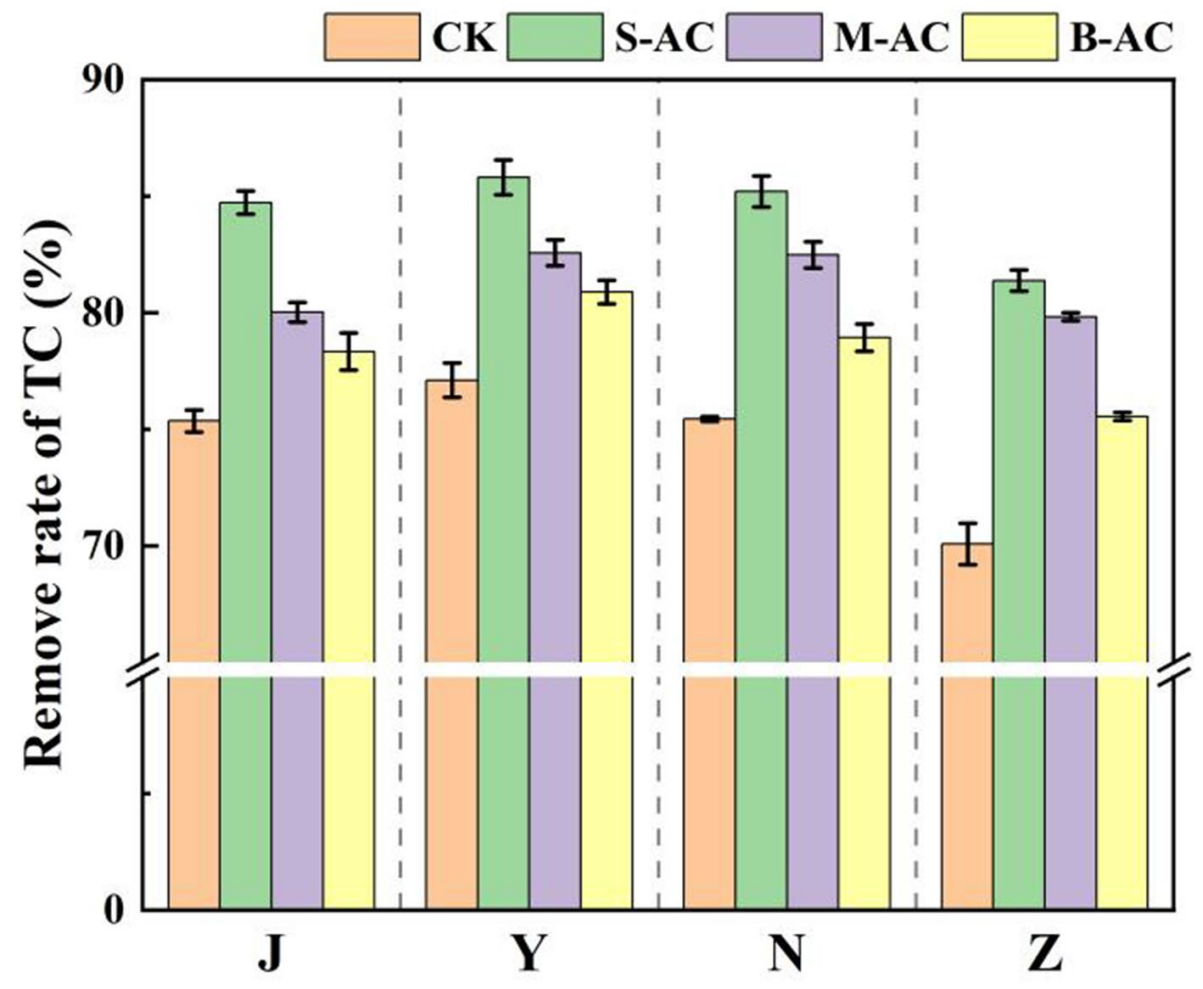
Remove rates of TC (30 d).

As shown in Figure 16, the dynamic changes in TC degradation for different treatment groups were basically the same, all showing a trend of gradually decreasing with the repair time. The application of all three types of activated carbon reduced the residual amount of TC in the organic fertilizer. Among them, the S-AC group had the best TC removal effect. Figure 17 further quantitatively compared the TC degradation rates of each group at 30 days. The results showed that the S-AC group achieved the highest degradation rate among the four types of organic fertilizers (chicken manure, sheep manure, cow manure, and pig manure), reaching 84.72, 85.81, 85.20, and 81.38% respectively, which was significantly better than the other treatment groups.

**Figure 18:**
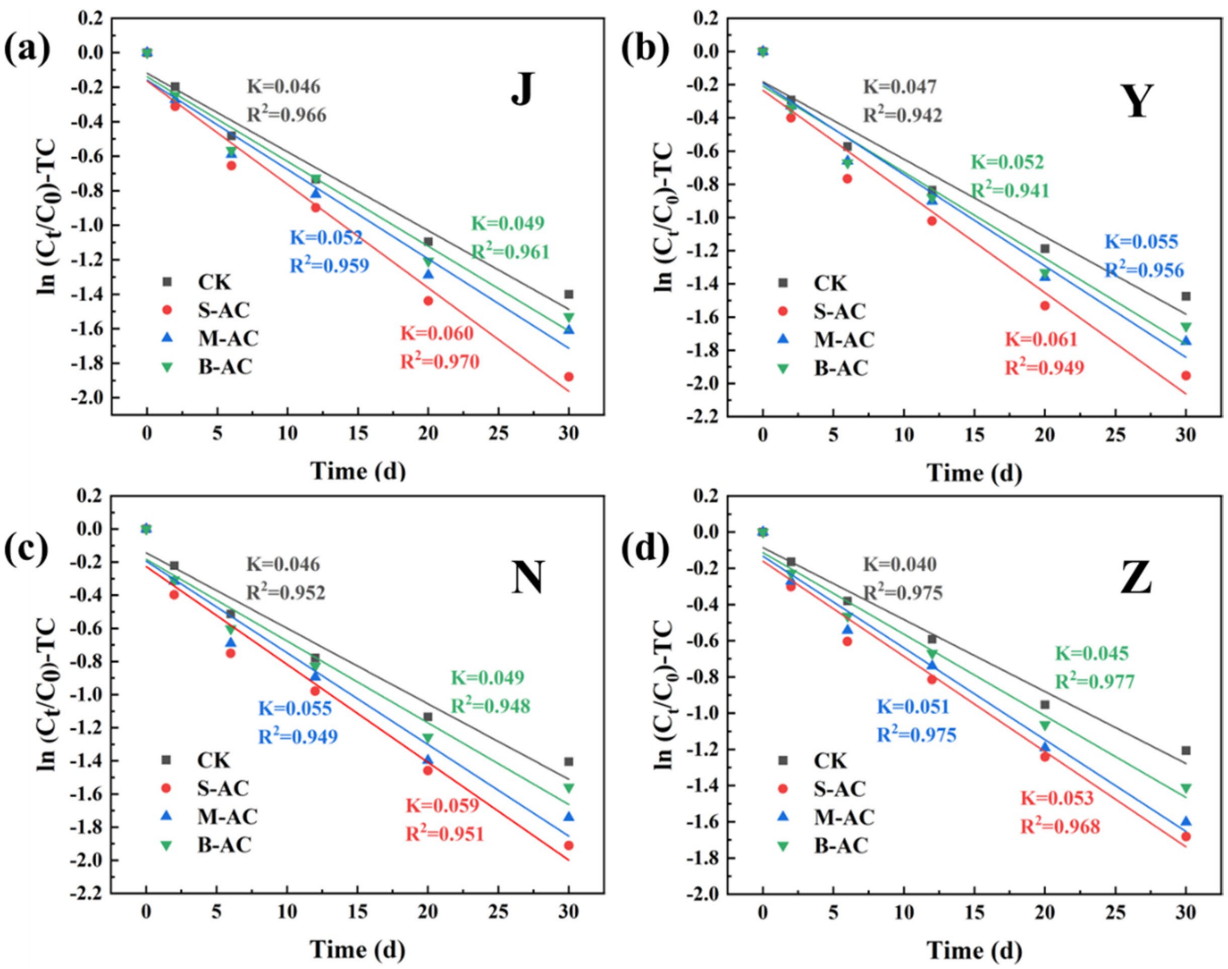
TC first-order kinetic fitting curve: **(a)** J group; **(b)** Y group; **(c)** N group; **(d)** Z group.

**Figure 19:**
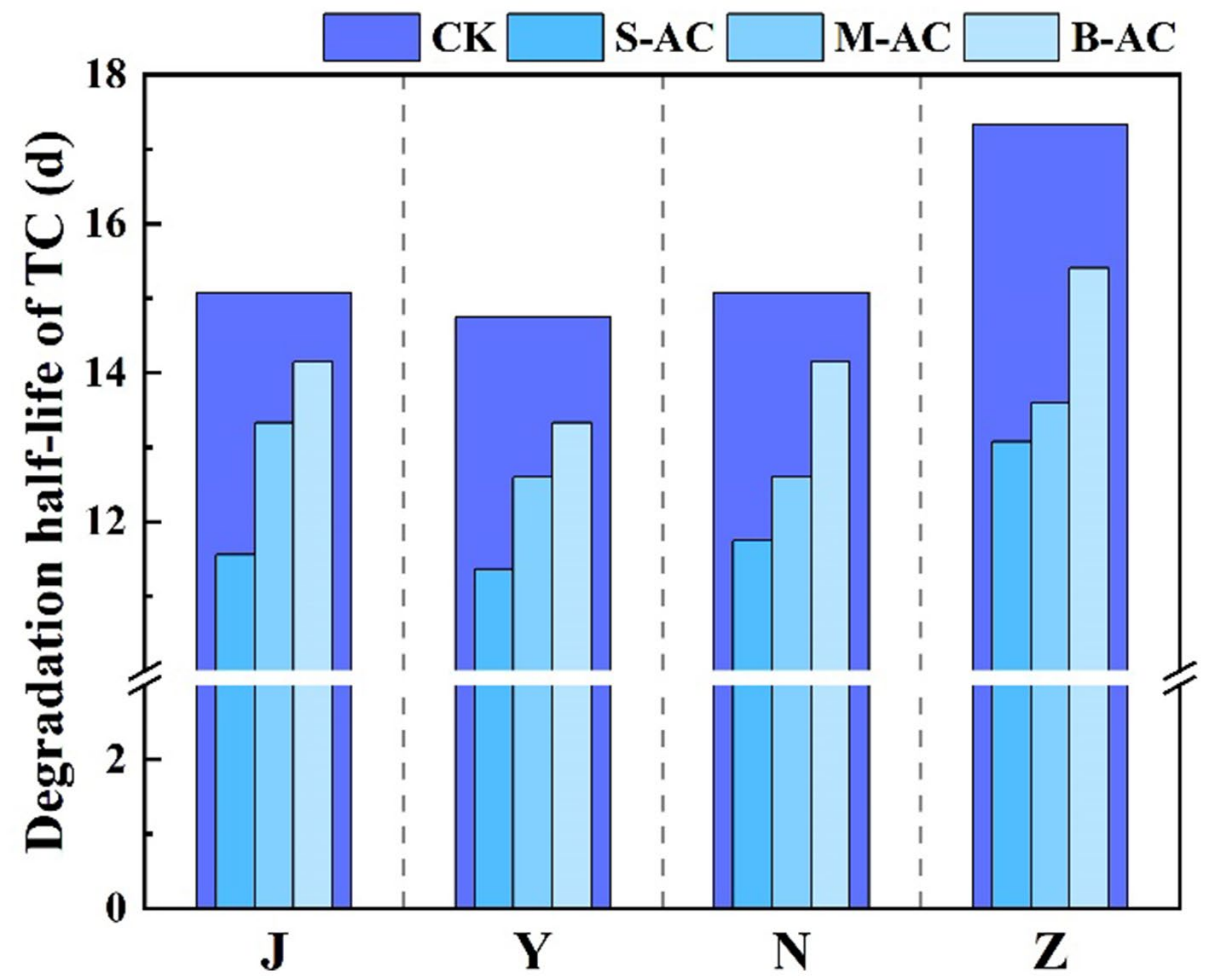
Half-life of TC degradation.

The degradation process of TC concentration over time was fitted using the first-order kinetic equation (Figure 18). The correlation coefficient R^2^ of all treatment groups exceeded 0.94, indicating that the degradation process of TC in the organic fertilizer follows the pseudo-first-order reaction kinetic equation. K represents the degradation rate during the cultivation process, and the K value reaches its maximum in the S-AC treatment group, suggesting that TC degradation is the fastest under the treatment with S-AC. The half-life t_1/2_ reflects the time required for the TC concentration in the organic fertilizer to decrease by half. As shown in Figure 19, the groups with added particle activated carbon have shorter half-life times than the control group. The addition of activated carbon, especially S-AC, can effectively promote the degradation process of TC in the organic fertilizer and increase the TC removal rate.

**Figure 20:**
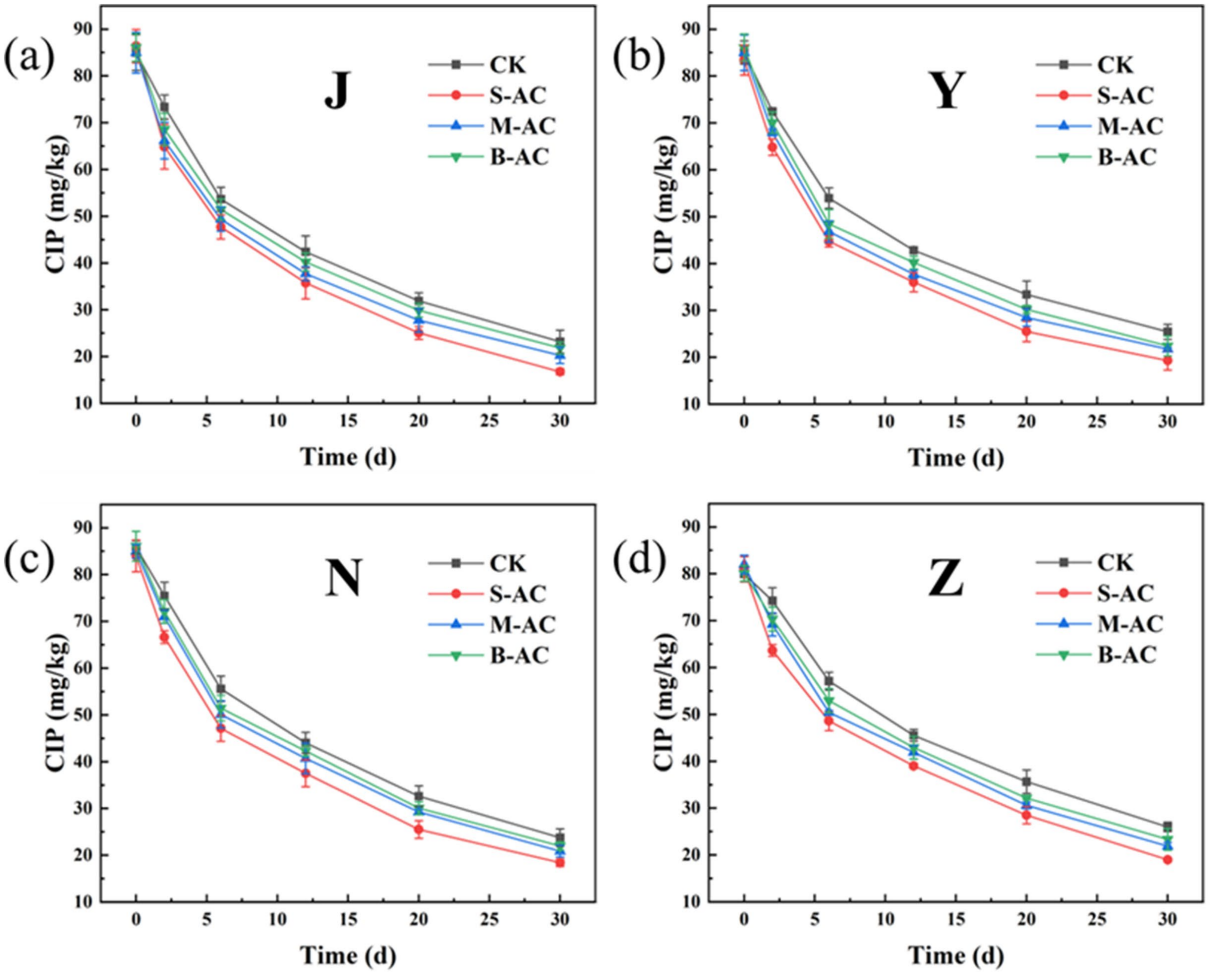
Changes in CIP concentration: **(a)** J group; **(b)** Y group; **(c)** N group; **(d)** Z group.

**Figure 21:**
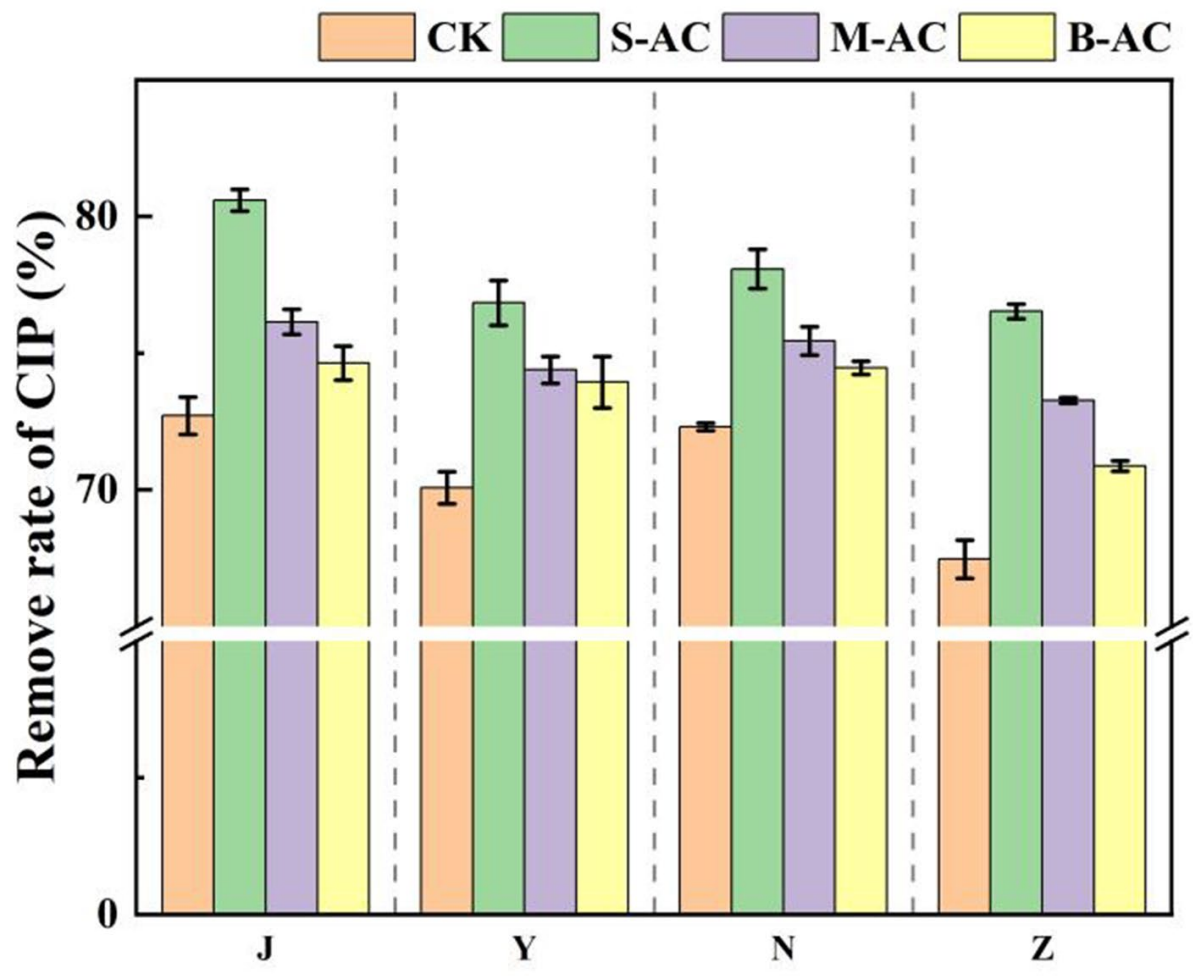
Remove rates of CIP (30 d).

As shown in Figure 20, similar to TC, the residual amount of CIP in each treatment group decreased with the extension of the restoration time, indicating that different activated carbon treatments could effectively promote the degradation of CIP. Among them, the degradation effect of the S-AC treatment group was the most significant. The degradation rate data after 30 days (Figure 21) showed that the CIP removal rate of the S-AC group in chicken manure, sheep manure, cow manure and pig manure reached 80.59, 76.84, 78.08 and 76.53% respectively, which were significantly higher than those of other treatment groups, indicating that small particle activated carbon has a stronger promoting effect on the degradation of CIP.

**Figure 22:**
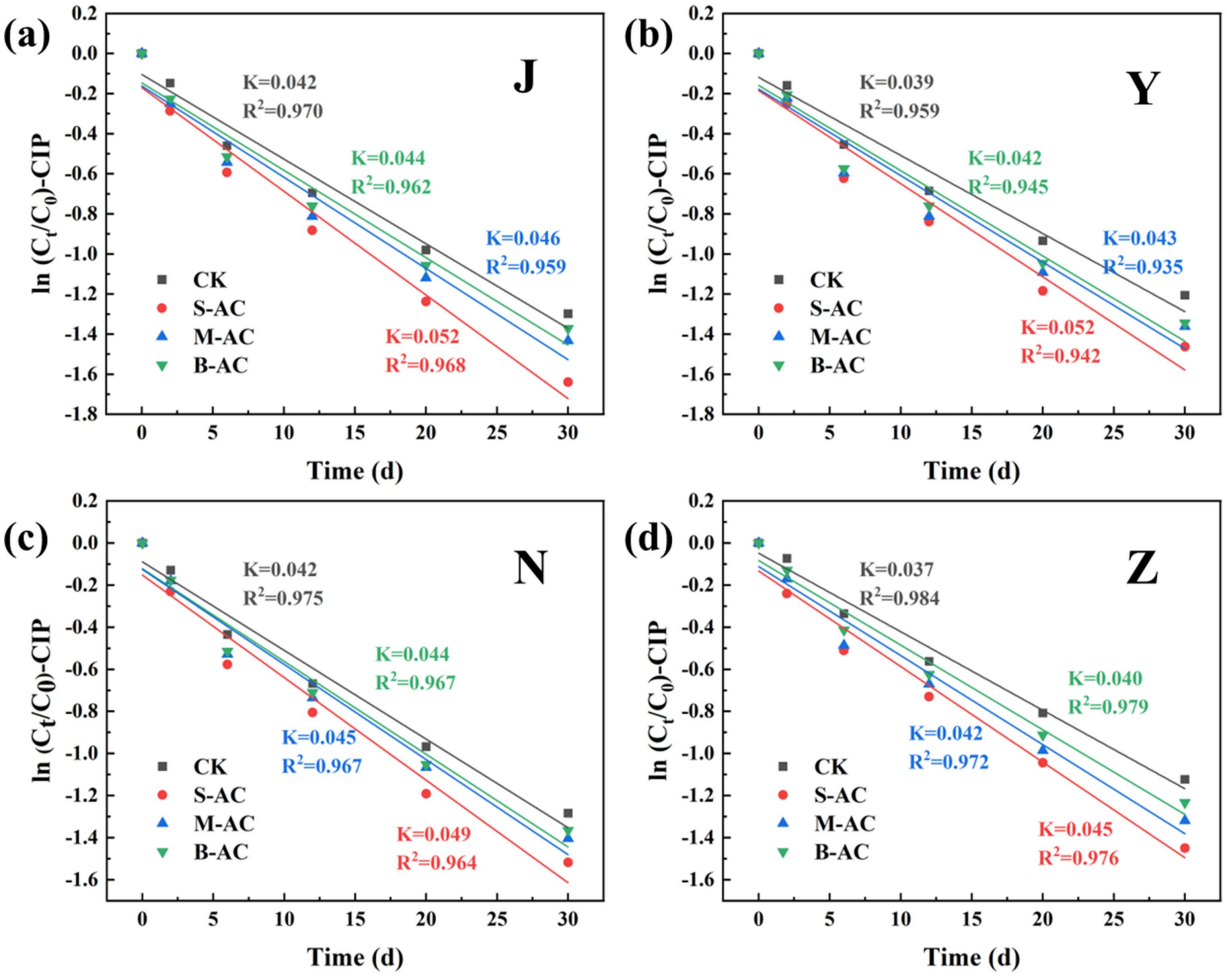
CIP first-order kinetic fitting curve: **(a)** J group; **(b)** Y group; **(c)** N group; **(d)** Z group.

**Figure 23:**
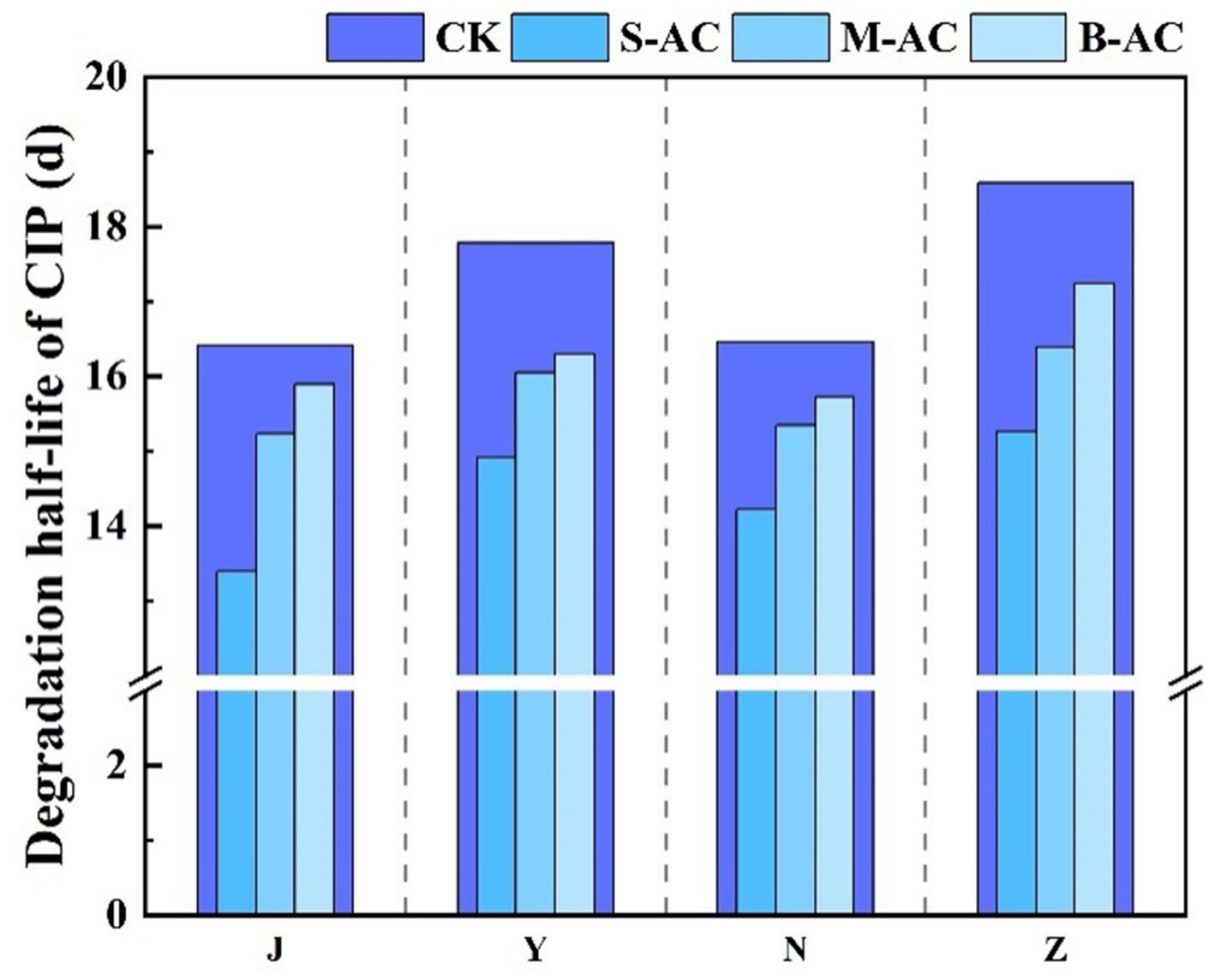
Half-life of CIP degradation.

The degradation process of CIP was also fitted using the first-order kinetic model (Figure 22). The goodness of fit (R^2^) of all treatment groups was higher than 0.93, indicating that the degradation of CIP also conforms to the characteristics of a pseudo-first-order reaction. The analysis of kinetic parameters showed that the degradation rate constant (K) of the S-AC group was the highest, indicating that the degradation rate of CIP was the fastest under this treatment. Additionally, the half-life (t_1/2_) analysis (Figure 23) revealed that the treatment groups with activated carbon addition significantly shortened the degradation period of CIP compared to the control group. Among them, the S-AC group had the shortest half-life, further confirming that S-AC can effectively shorten the degradation period of CIP and increase its removal rate.

## 4 Conclusion

This study systematically evaluated the effects of different particle sizes of activated carbon on the physical and chemical properties of organic fertilizers, the immobilization of heavy metals, and the degradation of antibiotics. The addition of particle activated carbon can increase the pH value of organic fertilizers and reduce the CEC value. Its fragmentation leads to an increase in organic matter in organic fertilizers, but has no significant effect on the EC value. Small particle activated carbon (S-AC) performed the best in the removal of heavy metals (total and available Cu and Zn) and the degradation of antibiotics (TC and CIP). The addition of activated carbon not only changed the speciation of heavy metals and reduced their bioavailability, but also accelerated the degradation of antibiotics. Future research can further optimize the addition ratio of activated carbon and modify the activated carbon to enhance the comprehensive benefits of pollution control.

## Data Availability

The original contributions presented in the study are included in the article/supplementary material, further inquiries can be directed to the corresponding author.
